# Responses of zinc recovery to temperature and mineral composition during sphalerite bioleaching process

**DOI:** 10.1186/s13568-017-0491-1

**Published:** 2017-10-23

**Authors:** Yunhua Xiao, Xueduan Liu, Jun Fang, Yili Liang, Xian Zhang, Delong Meng, Huaqun Yin

**Affiliations:** 1 College of Bioscience and Biotechnology and College of Agronomy, Hunan Agricultural University, Changsha, 410128 China; 2 School of Minerals Processing and Bioengineering, Central South University, Changsha, 410083 China

**Keywords:** Temperature, Original mineral compositions, Microbial community, Zinc leaching efficiency, Models

## Abstract

Temperature and energy resources (e.g., iron, sulfur and organic matter) usually undergo dynamic changes, and play important roles during industrial bioleaching process. Thus, it is essential to investigate their synergistic effects and the changes of their independent effects with simultaneous actions of multi-factors. In this study, we explored the synergistic effects of temperature and original mineral compositions (OMCs, energy resources) on the sphalerite bioleaching process. The microbial community structure was monitored by 16S rRNA gene sequencing technology and showed clear segregation along temperature gradients and Shannon diversity decreased at high temperature. On the contrary, the physicochemical parameters (pH and [Fe^3+^]) in the leachate were significantly affected by the OMCs. Interestingly, the influence of temperature on zinc recovery was greater at relatively simpler OMCs level, whereas the influence of OMCs was stronger at lower temperature. In addition, using [Fe^3+^], pH, relative abundances of dominant OTUs of microbial community and temperature as variable parameters, several models were constructed to predict zinc leaching efficiency, providing a possibility to predict the metal recovery efficiency under temperature change and variable energy resources.

**Electronic supplementary material:**

The online version of this article (doi:10.1186/s13568-017-0491-1) contains supplementary material, which is available to authorized users.

## Introduction

Seasonal and regional changes, accompanying changes of temperature and nutrient/energy resources, are regarded as two of main drivers regulating microbial diversity and function in natural ecosystem (Gilbert et al. 2012; Wang et al. 2016), e.g. marine ecosystem and forest soil ecosystem. However, due to the high richness and unculturable character of most species in the majority of natural ecosystems, the functions of microbes are still unclear and the importance of the change of community composition with temperature or nutrient increase/decrease is confusing (Streit and Schmitz 2004).

Bioleaching system is a relatively simpler ecosystem, since the species richness in this system is much lower than other ecosystems for its characters of low pH value and high heavy metal concentration. The dominant species in bioleaching systems are chemoautotrophic microbes, most of which has been isolated and identified, and their optimal growth conditions (pH, temperature and energy resource, etc.), genome sequencing and function were well studied (Goto et al. 2002; Mangold et al. 2011; Justice et al. 2014; Bonnefoy and Holmes 2012). Therefore, it is suitable to use the bioleaching system to predict the variation of function via the change of microbial community under the change of temperature and energy resources.

Temperature of the surface layer is variable affected by seasonal and regional conditions in industrial bioleaching system. Many studies have o been done and planned to reveal the effects of temperature on bioleaching process (Behrad Vakylabad 2011; Watling et al. 2016). Behrad Vakylabad et al. (2011) reported that copper recovery was 87.52% at 50 °C with moderately thermophilic microorganisms and was 34.55% at 35 °C with mesophilic microorganisms in chalcopyrite bioleaching. However, in these researches, single factor (temperature) was focused, whereas the differences of microbial community had been ignored at different temperature.

Studies (Basson et al. 2013; Mousavi et al. 2008; Shiers et al. 2014) found that, to some extent, energy resources (sulfur, iron or organic matter) could also enhance metal recovery. Mineral, which comprises sulfur and/or iron, is one kind of energy resource. Zhao et al. (2015) revealed that pyrite which had high concentration of iron enhanced the dissolution rate of chalcopyrite, which was consistent with Nazari et al. (2011). In addition, it consists of different minerals in natural mines, whereas the interaction between different minerals and the effects of multi-minerals on microbial community had always been neglected during bioleaching process.

There are three scientific questions in this study: (i) With simultaneous change of temperature and mineral composition, what was the responses of the microbial community, physicochemical parameters and metal recovery efficiency? (ii) What are the complex relationships among temperature, mineral composition, microbial community and zinc recovery? (iii) According to the known parameters, how to construct model to predict metal recovery efficiency? To explore these questions, in this study, four original mineral compositions were bioleaching at five different temperatures. 16S rRNA gene sequencing technology was used to monitor microbial community diversity and structure. Models were also constructed to predict zinc recovery efficiency using related parameters in bioleaching systems. The results gave us a better understanding of the influence of temperature and mineral composition on microbial community and metal recovery in bioleaching system, and suggested that the suitable shift of substrate (e.g., mineral composition and the concentration of ferric iron) and temperature enhanced metal recovery. These findings are meaningful in the industrial bioleaching systems and provide possible methods for predicting the ecosystem function following the change of temperature and energy resources.

## Materials and methods

### Bacterial cultures, minerals, bioleaching treatments and Illumina sequencing

In this study, the bacterial culture was enriched as described in our previous study (Xiao et al. 2015) and supplemental materials. Three minerals, sphalerite, pyrite and chalcopyrite, were utilized and the compositions were shown in Additional file 1: Table S1.

Bioleaching treatments, including four mineral composition treatments, which were sphalerite only (S), added with pyrite (SP, w/w, 1:1), with chalcopyrite (SC, w/w, 1:1) and with both (SPC, w/w/w, 1:1:1), were conducted at five different temperature treatments (30, 35, 40, 45 and 50 °C), respectively. All treatments (4 mineral treatments × 5 temperature treatments) were carried out in septuplicate. The physicochemical parameters, including pH, redox potential (ORP), the concentration of dissolved ferrous iron, total iron, copper ion, zinc ion and sulfate ion in the solution were monitored and a flask of each experimental group was removed for DNA extraction on day 6, 12, 21 and 30 (Xiao et al. 2015). Physicochemical parameters analyses, cells collection, DNA extraction, 16S rRNA gene Illumina sequencing and data processing were carried out as described previously (Xiao et al. 2015) and also see a detailed description in supplementary materials. The raw data of 16S rRNA gene Illumina sequencing has been submitted to sequence read archive (SRA) of NCBI database and the accession number was SUB2165181.

### Statistical analysis

Microbial community diversity was evaluated by Shannon diversity index (Shannon 1948). Differences were determined by a one-way analysis of variance followed by a Least Significant Difference test. Detrended correspondence analysis (DCA) was conducted to compare all samples of different bacterial community structures (at OTU level) and different OMCs, and principal component analysis (PCA) was conducted to compare all samples of different physicochemical parameters, All the analyses were performed in R v. 3.1.12 with the vegan (v. 1.11-3) packages, Origin v 8.1, PermutMatrixEN or online (http://ieg.ou.edu/).

To explore relationships among temperature, physicochemical parameters, the OMCs, microbial community structure, microbial diversity (Shannon diversity index) and function (zinc leaching efficiency), Pearson correlation test and Partial Least Squares Path Modeling (PLS-PM) was carried out (Sanchez et al. 2015). The whole physicochemical parameters were represented by the values of PC1 and PC2 (the top two axes) of PCA, and the OMCs and microbial community structure were represented by the values of DCA1 and DCA2 (the top two axes) by DCA. Three models by PLS-PM were conducted using temperature, the OMCs, physicochemical parameters/community structures/community diversity at four different stages, and zinc leaching efficiency.

### Prediction model of zinc leaching efficiency

Multiple linear regression analysis (MLR) and neural network were applied to construct the models to predict zinc leaching efficiency in response to the changes of temperature, the OMCs, ferric iron, pH value and dominant OTUs. MLR is a simple model, while neural network is a method to capture and model the complex interactions between zinc leaching efficiency and the various parameters (Larsen et al. 2012). In this study, we use two packages, neuralnet (v. 1.33) and nnet (v. 7.3-10) in R software. The best-fitted equations based on the optimality criteria were then used for the prediction. In the formula search, data from 50 randomly selected samples of total 80 samples were used for model training. After gaining the best-fitted equation, the remaining 30 samples were imported to validate this equation. The random samples were reshuffled 10 times.

The goodness of fit with models was assessed by the coefficient of determination (R^2^) and Akaike information criterion (AIC) (Zhou et al. 2016). The coefficient of determination is defined as: R^2^ = 1 − SSR/TSS, where SSR is the sum of squares of residuals and TSS is the total sum of squares; The AIC is defined as: AIC = − 2 × ln(L) + 2n, where L is the probability of the data given a model and n is the number of parameters (Zhou et al. 2016). Better model fits with greater R^2^ value (smaller SSR value) and smaller AIC value.

## Results

### Effects of temperature and mineral composition on zinc recovery

**Fig. 1:**
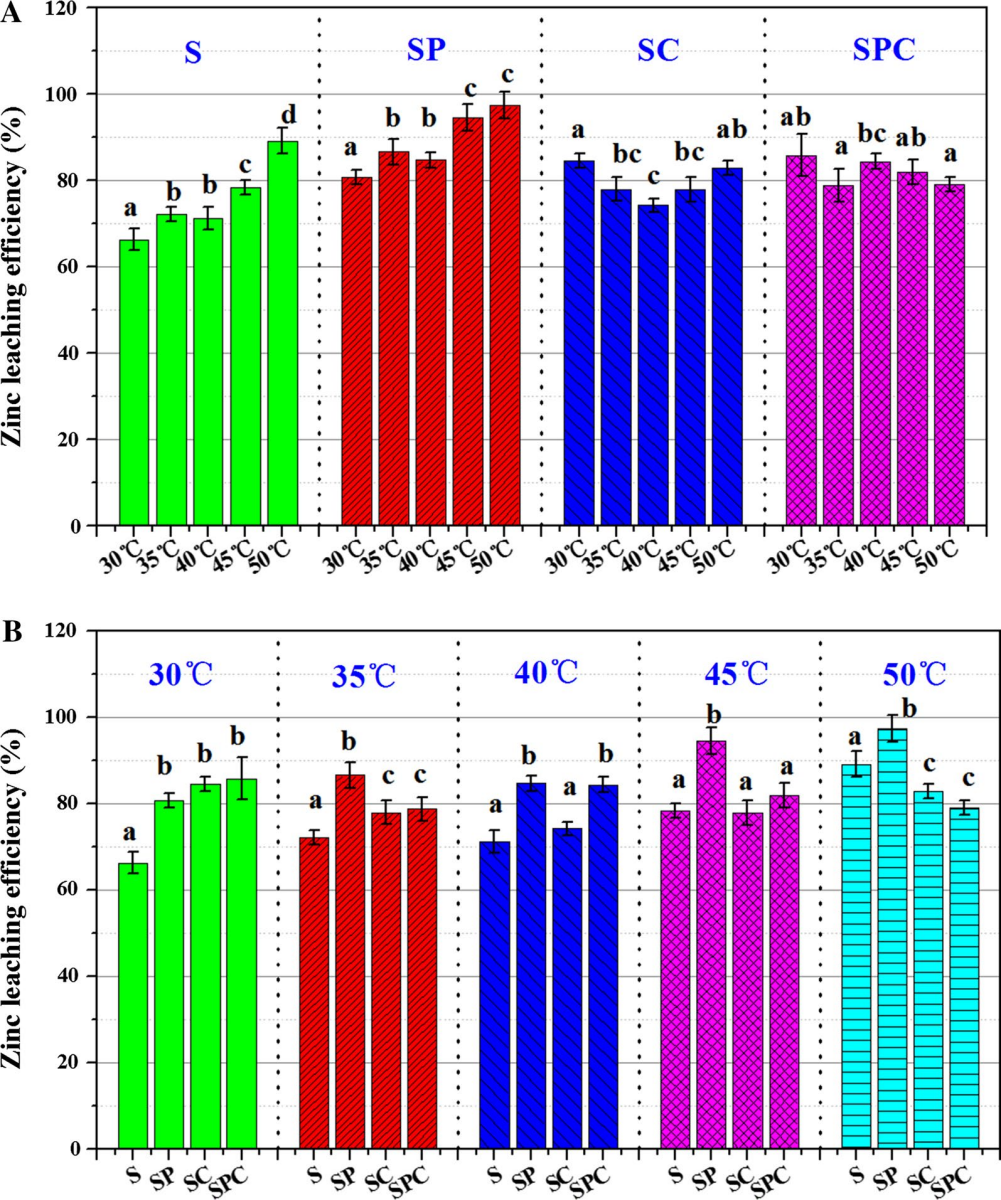
Zinc leaching efficiency on day 30 under different temperature (**A**) and mineral composition (**B**)

The rate of mineral decomposition and zinc leaching efficiency differed among treatments at different stages (Additional file 1: Table S2). On day 30, zinc leaching efficiency was 66.3% at 30 °C, 72.2% at 35 °C, 71.2% at 40 °C, 78.2% at 45 °C and 89.1% at 50 °C in S group, and it was 80.7% at 30 °C, 86.5% at 35 °C, 84.6% at 40 °C, 94.5% at 45 °C and 97.2% at 50 °C in S group (Fig. 1A), which indicated that higher temperature could lead to higher zinc leaching efficiency. It was not consistently with that in SC and SPC groups. In SC group, zinc leaching efficiency was the lowest at 40 °C (74.1%) and was the highest at 30 (84.4%) and 50 °C (82.9%). In SPC group, zinc leaching efficiency was the highest at 30 °C (85.8%) and was the lowest at 50 °C (78.9%). Figure 1B showed that zinc leaching efficiency was higher in SP group than that in S group at 5 different temperature gradients, however, it was higher in SC and SPC group than that in S group at lower temperature (30, 35 and 40 °C), and was lower than that in S group at 50 °C.

### Variation of physicochemical parameters during sphalerite bioleaching process

**Fig. 2:**
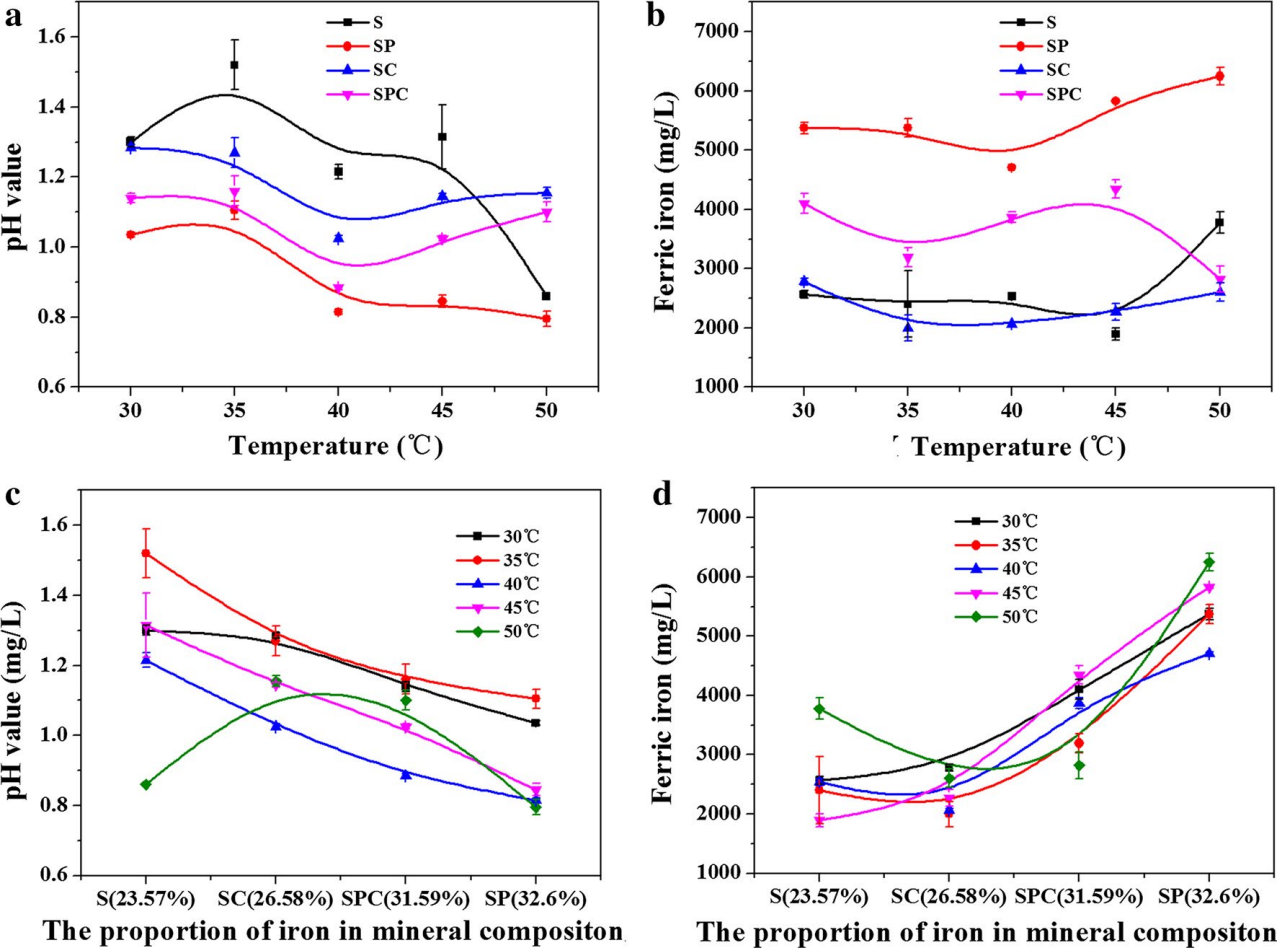
pH value and the concentration of ferric iron on day 30 under different temperature (**a** pH; **b** ferric iron) and different mineral composition (**c** pH; **d** ferric iron)

The variations of physicochemical parameters, e.g., pH, ORP, concentration of Fe^3+^, were shown in Additional file 1: Table S3. In all treatments, the pH value and the concentration of Fe^2+^ decreased gradually, while ORP and the concentrations of Fe^3+^, SO_4_^2−^, Zn^2+^ and Cu^2+^ increased gradually. Figure 2a, b showed that the variation trends of pH value and the concentration of ferric iron were not regular with an increase of temperature under different OMCs on day 30. In S group, the lowest pH value was 0.86 at 50 °C, and the highest was 1.52 at 35 °C; in SP group, the lowest was 0.795 at 50 °C, and the highest was 1.105 at 35 °C; in SC group, the lowest was 1.025 at 30 °C, and the highest was 1.285 at 35 °C; in SPC group, the lowest was 0.885 at 40 °C, and the highest was 1.16 at 35 °C. In S group, the highest ferric iron concentration was 3.78 g/L at 50 °C, and the lowest was 2.40 g/L at 45 °C; in SP group, the highest was 6.25 g/L at 50 °C, and the lowest was 4.71 g/L at 40 °C; in SC group, the highest was 2.78 g/L at 35 °C, and the lowest was 2.00 g/L at 40 °C; in SPC group, the highest was 4.35 g/L at 45 °C, and the lowest was 2.82 g/L at 50 °C. Figure 2c, d showed that the pH value showed decreasing trend and the concentration of ferric iron showed increasing trend with the increase of iron proportion in different OMCs under different temperature (except for 50 °C) on day 30.

**Fig. 3:**
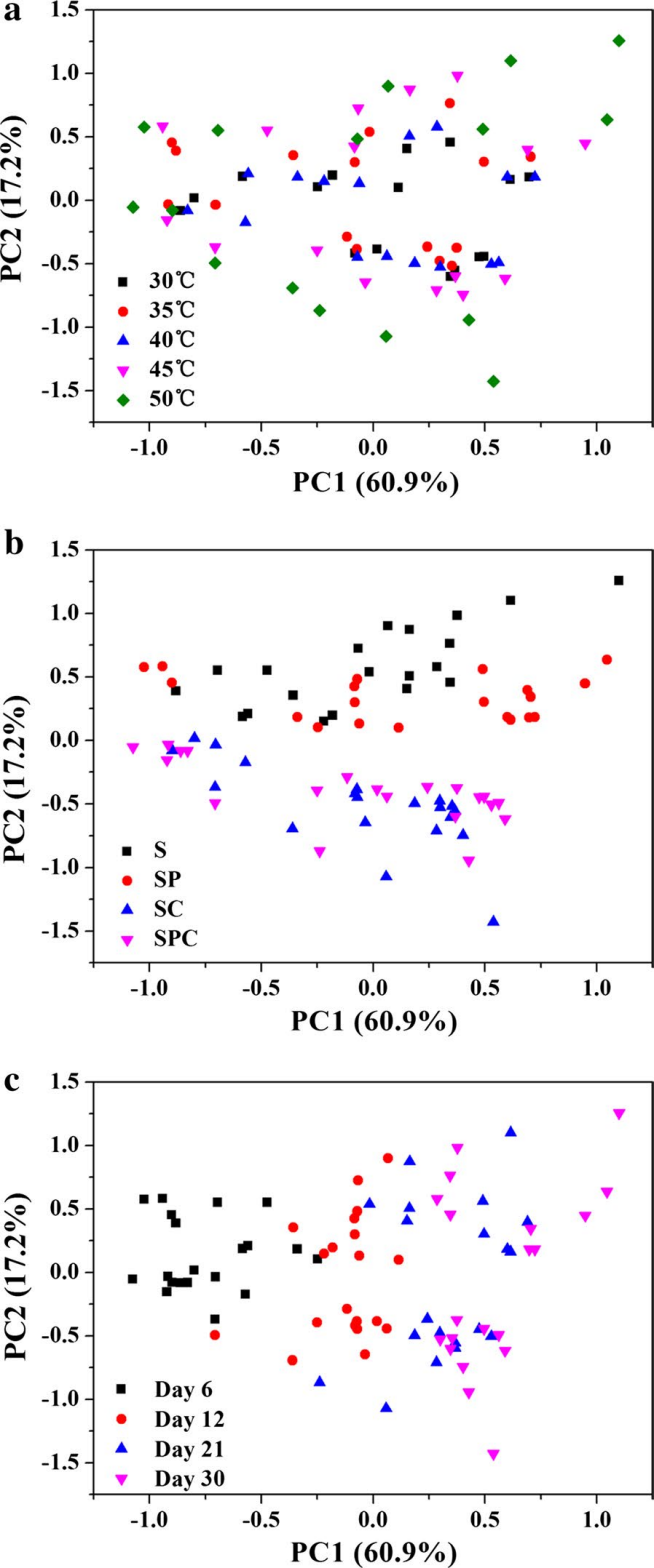
Plot of physicochemical parameters of all samples under different temperature (**a**), mineral composition (**b**) and stages (**c**) using principal component analysis (PCA)

PCA was conducted to compare the physicochemical parameters of all treatments (Fig. 3). It showed that the OMCs (Fig. 3b), rather than temperature (Fig. 3a), had a significant effect on physicochemical parameters. In addition, the physicochemical parameters were also significantly different between bioleaching stages (day 6, 12, 21 and 30, Fig. 3c).

### Overview of microbial community

In order to reveal the effects of temperature and the OMCs on the microbial community during the sphalerite bioleaching process, 16S rRNA gene sequencing was conducted. After clustering at 97% sequence identity, 405 OTUs were identified in this study. Among these, 394 OTUs (97.28%) were classified in the domain *Bacteria*. Within the domain Bacteria, the dominant phyla were *Proteobacteria*, *Firmicutes* and *Nitrospirae*, which accounted for above 84.3% of all reads (Additional file 1: Figure S1). Within the domain *Archaea*, the dominant phylum was *Euryarchaeota*, which accounted for about 0–15.67% in this study. *Acidithiobacillus* (1.72–93.86%) and *Acidiphilium* (0–5.59%) were the dominant genera within the phylum *Proteobacteria*, *Sulfobacillus* (0.28–93.53%) within phylum *Firmicutes*, *Leptospirillum* (0–74.05%) within the phylum *Nitrospirae*, and *Ferroplasma* (0–15.96%) within the phylum *Euryarchaeota* (Additional file 1: Figure S2). In addition, the unclassified genera were accounted for 0–22.13%, which was mostly affiliated with *Proteobacteria*.

### Effects of temperature and mineral composition on microbial community structure and diversity

Comparison of the taxonomic populations among the 20 treatments was conducted to determine how populations, structure and diversity responded to the different temperature and mineral compositions.

We compared the differences of microbial compositions at genera level (Additional file 1: Figure S2) and found that relative abundances of some genera were affected by the OMCs or temperature. It showed that, compared with S group, high abundance was in SP, SC and SPC groups. In S group, *Leptospirillum* accounted for a certain proportion (> 1%) at 30 and 35 °C on day 30. In other three groups, compared with 30, 35, 40 and 45 °C, few *Leptospirillum* was detected at 50 °C. Higher relative abundances of *Sphingobacterium* were at 45 or 50 °C than that at 30, 35 and 40 °C. Certain abundances of *Alicyclobacillus* were detected at 40 °C in S and SP group. *Acidiphilium* were only detected at 30 °C at the initial stage (day 6 and 12).

**Fig. 4:**
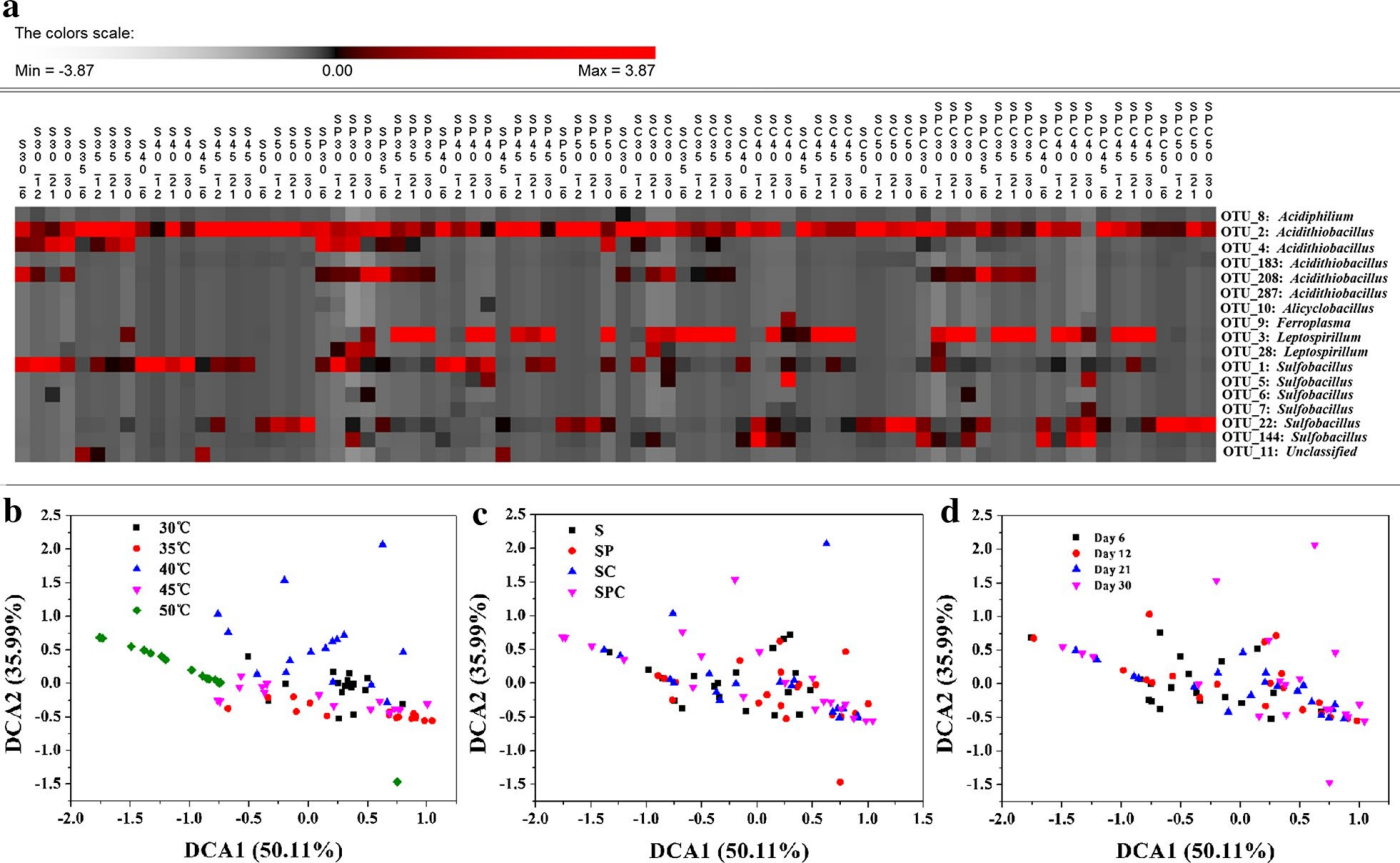
Polts of microbial community composition at OTU level (**a**) and microbial community structure under different temperature (**b**), mineral composition (**c**) and stages (**d**) using detrended correspondence analysis (DCA)

Microbial compositions changed more obviously at the OTU level (Fig. 4a). For example, although six OTUs, including OTU_1, OTU_5, OTU_6, OTU_7, OTU_22 and OTU_144, were all affiliated to *Sulfobacillus*, their responses to the temperature and mineral composition were different. With the effects of temperature, OTU_1 was more abundant at 30 and 40 °C, OTU_5 and OTU_7 were more abundant at 40 °C, OTU_6 was more abundant at 30 °C, OTU_22 was more abundant at 50 °C; With the effects of mineral composition, OTU_1 was more abundant in S and SP, OTU_6 was more abundant in S, SP and SPC groups, and OTU_5, OTU_7 and OTU_144 were more abundant in SC and SPC groups. The similarity was found in *Acidithiobacillus*. OTU_2 was showed no significant different in all treatments, but OTU_4 and OTU_208 were more abundant at 30 and 35 °C, and OTU_208 were more abundant in SP and SPC groups. Results of NCBI blast (nr/nt database) showed the taxa of the top 17 OTUs (Additional file 1: Figure S3), indicating that the OTUs, affiliated to the same genus, might be different species or be different strains of the same genus. For example, OTU_1 belonged to *S. thermotolerans* strain Kr1 (98%), while OTU_6 belonged to *S. acidophilus* strain DSM 10332 (96%).

Due to the effects of temperature and the OMCs on OTUs, the Shannon diversity was also influenced (Additional file 1: Table S4). On the one hand, it has been found that relatively higher temperature would lead to lower Shannon diversity index. For example, in SP group on day 21, the Shannon diversity index was 2.136 at 30 °C, 1.307 at 35 °C, 1.406 at 40 °C, 1.176 at 45 °C and 0.835 at 50 °C. On the one hand, the Shannon diversity index was different under different OMCs. For example, at 30 °C on day 21, it was 1.257 in S group, 2.136 in SP group, 1.807 in SC group and 1.312 in SPC group, while at 50 °C on day 21, it was 0.849 in S group, 0.835 in SP group, 0.845 in SC group and 0.910 in SPC group. However, it seemed that the change of the OMCs had a certain but not a regular effect on Shannon diversity index.

To explore the differences of the whole microbial community structure under different temperature and OMCs, DCA was conducted (Fig. 4). It showed that samples at different temperature could separate from each other, except some samples at 35 and 45 °C, while samples in different OMCs and at different bioleaching stage could not separate from the other groups.

### Relationship among temperature, mineral composition, physicochemical parameters, microbial community structure, microbial diversity and zinc leaching efficiency

**Fig. 5:**
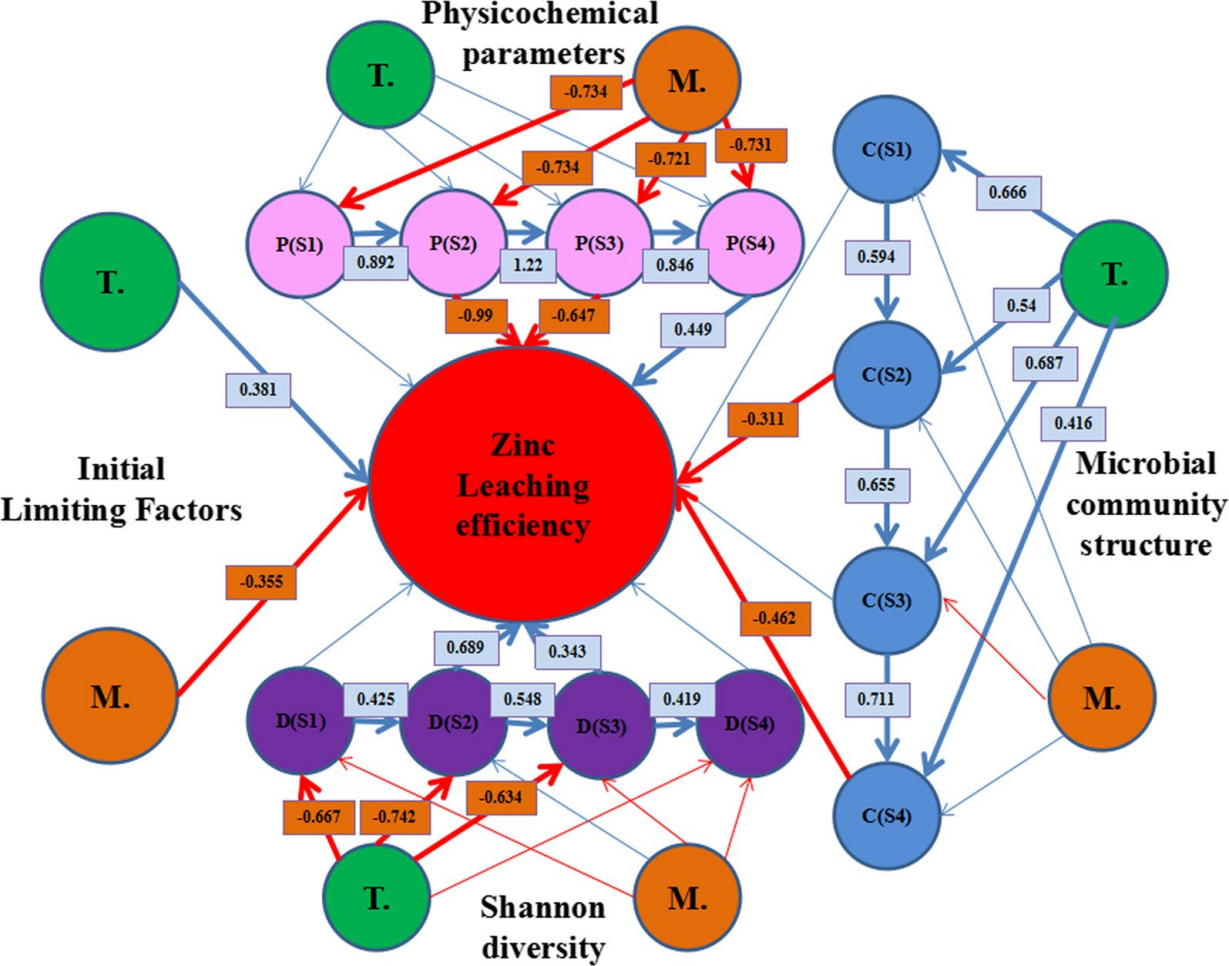
The direct and indirect effects of temperature and the original mineral composition on zinc leaching efficiency. The effects of temperature, mineral composition and physicochemical parameters (model 1)/microbial community structure (model 2)/microbial diversity (model 3) on zinc leaching efficiency, explored with Partial Least Squares Path Modeling (PLS-PM). Mineral composition and microbial community structure were represented by the top two axes (DCA1 and DCA2) of DCA. The whole physicochemical parameters was represent by the values of PC1 and PC2 (the top two axes) of PCA. The blue line represented the positive correlation, and the red line represented the negative correlation. The bold line showed the relation was significant (p < 0.05) or marginally significant (p < 0.1) and the regular line showed the relation was no significant. Models were assessed using goodness of fit (GoF) statistic. The GoFs for model 1–3 were 0.642, 0.599 and 0.642, respectively

To explore the relationships among temperature, the OMCs, physicochemical parameters, microbial community structure, microbial diversity and zinc leaching efficiency in bioleaching system at the whole level, Partial Least Squares Path Modeling (PLS-PM) was used and the result was shown in Fig. 5. In this model, physicochemical parameters, including pH, [Fe^2+^], [Fe^3+^], ORP, [Cu], [SO_4_^2−^] and [Zn], were represented by the values of PC1 and PC2 (the top two axes) of PCA. Shannon diversity index represented microbial diversity. The OMCs and microbial community structure were represented by the values of DCA1 and DCA2 (the top two axes) of DCA. Zinc leaching efficiency was important in sphalerite bioleaching system, and thus it still represented the function of the microbial community. In addition, the physicochemical parameters, microbial community structure and microbial diversity were utilized at the four stages (day 6, 12, 21 and 30), respectively. Temperature (*r* = 0.381) and the OMCs (*r* = − 0.355) were both correlated to zinc leaching efficiency. Zinc leaching efficiency had significant relationships (*r* > 0.300, *p* < 0.05) with the physicochemical parameters and Shannon diversity index on day 12 and 21 also, and microbial community structure on day 6 and 30. This model also showed that temperature was significantly influenced microbial community structure and Shannon diversity index, while had no significant effects on physicochemical parameters. On the contrary, the OMCs was significantly correlated to physicochemical parameters, while had no significant effects on microbial community structure and Shannon diversity index.

Besides, in order to better understand the effects of relative abundance of each OTU and each physicochemical parameter on zinc leaching efficiency at the final stage (on day 30), Pearson correlation tests were conducted at the four stages, respectively (Table 1, Additional file 1: Table S5 and Table 2). Among these 405 OTUs, zinc leaching efficiency was significantly correlated to 8 OTUs on day 6, to 3 OTUs on day 12, to 23 OTUs on day 21 and to 47 OTUs on day 30 (Additional file 1: Table S5). Among the 17 dominated OTUs, OTU_1 (on day 6, 12 and 21), OTU_4 (on day 21) and OTU_6 (on day 21) were significantly (*p* < 0.05) or marginally significantly (*p* < 0.1) and negatively correlated to zinc leaching efficiency, and OTU_2 (on day 6 and 12) and OTU_5 (on day 6) were (p < 0.05) or marginally significantly (*p* < 0.1) and positively correlated to zinc leaching efficiency. Ferrous iron on day 6 and ferric iron on day 21 and 30 was significantly positive correlated to zinc leaching efficiency. The pH value and sulfate ion was also significantly correlated to zinc leaching efficiency. However, the correlation between pH value and zinc leaching efficiency was positive on day 6 and was negative on day 21 and 30.

**Table 1:** The correlation between zinc leaching efficiency on day 30 and the dominant OTU at different stages

OTU	Day 6		Day 12		Day 21		Day 30	
	r	p	r	p	r	p	r	p
OTU_1	− *0.384*	*0.095*	− *0.435*	*0.055*	− *0.538*	*0.014*	− 0.304	0.192
OTU_2	*0.380*	*0.098*	*0.457*	*0.043*	0.197	0.405	− 0.217	0.359
OTU_3	− 0.101	0.671	0.230	0.329	0.091	0.704	0.357	0.122
OTU_4	− 0.071	0.768	− 0.177	0.456	− *0.543*	*0.014*	− 0.337	0.147
OTU_5	*0.509*	*0.022*	0.184	0.437	0.277	0.237	− 0.026	0.915
OTU_6	0.000	1.000	0.110	0.644	− *0.438*	*0.053*	0.171	0.471
OTU_7	0.207	0.381	0.215	0.362	0.128	0.591	0.075	0.753
OTU_8	0.220	0.352	− 0.024	0.920	− 0.337	0.146	0.312	0.180
OTU_9	0.363	0.116	0.320	0.169	− 0.136	0.568	− 0.141	0.555
OTU_10	0.070	0.768	0.152	0.523	− 0.263	0.263	0.120	0.613
OTU_11	− 0.217	0.359	− 0.130	0.584	− 0.327	0.159	0.000	1.000
OTU_22	0.164	0.489	0.070	0.769	0.261	0.267	0.153	0.519
OTU_28	− 0.002	0.992	0.239	0.311	0.164	0.490	0.001	0.997
OTU_144	0.140	0.556	− 0.172	0.469	0.047	0.843	0.102	0.670
OTU_183	0.118	0.620	0.346	0.135	0.137	0.565	− 0.003	0.990
OTU_208	− 0.067	0.780	− 0.262	0.264	− 0.010	0.966	− 0.103	0.667
OTU_287	0.024	0.920	− 0.298	0.201	0.181	0.446	0.043	0.856

Significant (*p* < 0.05) and marginally significant (*p* < 0.1) differences were indicated in italics

**Table 2:** The correlation between zinc leaching efficiency on day 30 and individual physicochemical parameter at different stages

Physicochemical parameters	Day 6		Day 12		Day 21		Day 30	
	r	p	r	p	r	p	r	p
pH	*0.492*	*0.028*	− 0.180	0.449	− *0.616*	*0.004*	− *0.814*	*0.000*
ORP	− 0.003	0.989	− 0.075	0.755	0.274	0.242	0.410	0.073
Fe^2+^	*0.455*	*0.044*	− 0.035	0.883	− 0.250	0.287	− 0.216	0.360
Fe^3+^	− 0.059	0.804	0.208	0.380	*0.543*	*0.014*	*0.744*	*0.000*
SO_4_^2−^	0.407	0.075	*0.592*	*0.006*	*0.745*	*0.000*	*0.786*	*0.000*
Zn^2+^	0.397	0.083	0.293	0.211	0.145	0.541	0.068	0.777
Cu^2+^	− 0.130	0.586	− 0.122	0.610	− 0.094	0.694	− 0.088	0.712

Significant differences (*p* < 0.05) were indicated in italics

### Model construction to predict zinc leaching efficiency

In this study, we explored the effect of temperature and the OMCs on sphalerite bioleaching, thus it was important whether temperature and the OMCs could predict zinc leaching efficiency at different stages. Multivariate linear regression was used to construct the predicting model (Table 3). The results showed that the models with temperature and the OMCs as the variable parameters could predict zinc leaching efficiency on day 6 (*R* ^2^ = 0.443, *p* = 0.006) and on day 30 (*R* ^2^ = 0.512, *p* = 0.002), but could not (*p* > 0.1) on day 12 and 21.

**Table 3:** Multiple regression analysis between zinc leaching efficiency and mineral composition on sampling time

Time	Formula	R^2^	p
Day 6	y = 2.800 + 0.816x_1_ + 721.911x_2_ − 741.258x_3_	0.443	0.006
Day 12	–	0.147	> 0.1
Day 21	–	0.038	> 0.1
Day 30	y = 62.266 + 0.479x_1_ + 462.157x_2_ − 470.054x_3_	0.512	0.002

Zinc leaching efficiency was indicated by “y” (unit: %), temperature was indicated by “x_1_” (unit: °C) and mineral composition was indicated by “x_2_” (DCA1) and “x_3_” (DCA2)

**Fig. 6:**
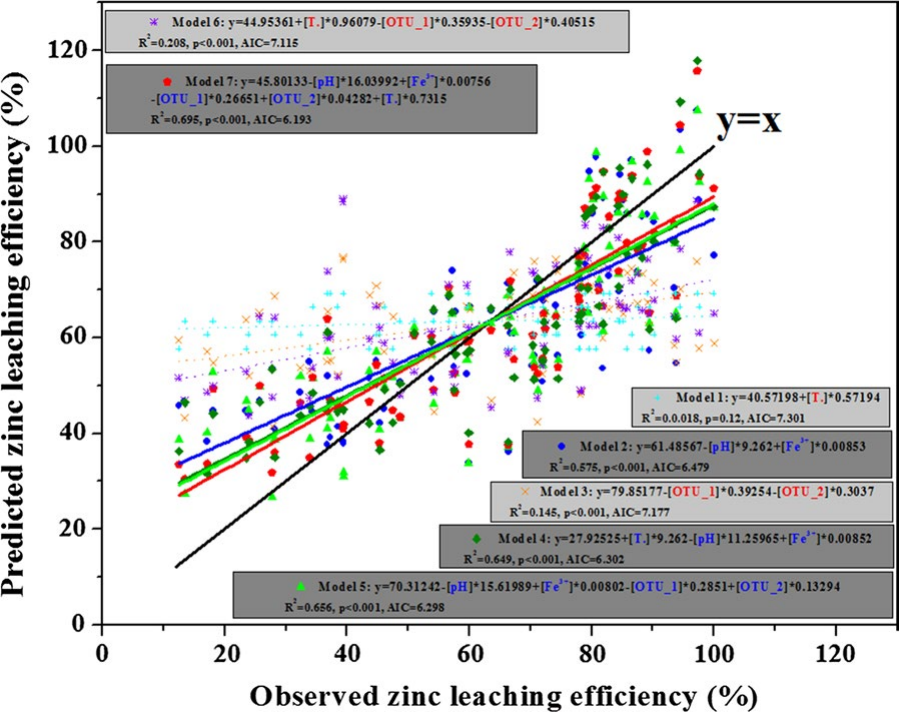
Models to predict zinc leaching efficiency using multiple linear regression analysis. The variable parameters were temperature, the concentration of ferric iron, pH value, OTU_1 (98% similarity to *S. thermotolerans* Kr1) or OTU_2 (98% similarity to *A. caldus* KU)

Through the above results, we know zinc leaching efficiency was significantly associated with microbial community, physicochemical parameters, temperature and the OMCs. Therefore, we could choose some of them to construct models to predict zinc leaching efficiency by multivariate linear regression. Firstly, considering that OMCs changed during bioleaching process, physicochemical parameters were significantly affected by mineral composition and the directly related reaction might be sulfur/iron oxidization, pH and the concentration of Fe^3+^ were chosen to represent the OMCs. Secondly, microbial community was complex, while the key OTUs might be relatively stable. OTU_1 (98% similarity to *S. thermotolerans* Kr1) and OTU_2 (98% similarity to *A. caldus* KU) were two of the most dominant OTUs and they were significantly correlated to zinc leaching efficiency, therefore, they were chosen to represent the microbial community. Seven multivariate linear regression models were constructed with 80 samples (Fig. 6). The independent variables were temperature in model 1; pH and the concentration of Fe^3+^ in model 2; OTU_1 and OTU_2 in model 3; temperature, pH and the concentration of Fe^3+^ in model 4; temperature, OTU_1 and OTU_2 in model 5; pH, the concentration of Fe^3+^, OTU_1 and OTU_2 in model 6; all factors in model 7. Among these models, model 2 (R^2^ = 0.575, p < 0.001, AIC = 6.479), 4 (*R* ^2^ = 0.649, *p* < 0.001, *AIC* = 6.302), 5 (*R* ^2^ = 0.656, *p* < 0.001, AIC = 6.298) and 7 (R^2^ = 0.695, p < 0.001, AIC = 6.193) could well predict zinc leaching efficiency, on the contrary, model 1 (*R* ^2^ = 0.018, *p* = 0.12, *AIC* = 7.301), 3 (*R* ^2^ = 0.145, *p* < 0.001, *AIC* = 7.177) and 6 (*R* ^2^ = 0.208, *p* < 0.001, *AIC* = 7.115) could not. It suggested that the models (model 7) with these five parameters were better fits.

**Fig. 7:**
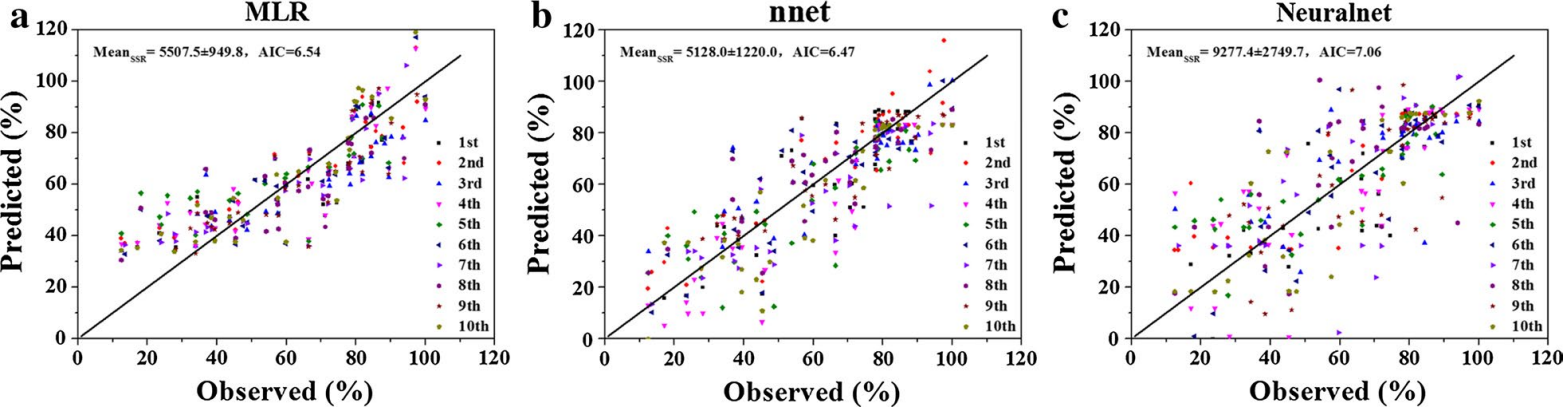
Comparison of three models to predict zinc leaching efficiency. **a** MLR model; **b** nnet model; **c** neuralnet model. 50 random samples were used to construct the models, and then the remaining 30 samples were predicted (reshuffled 10 times)

Considering the MLR models were too simple to predict, we compared this method with two kinds of neural network (neuralnet and nnet packages). Using the three constructed models with 50 samples, we predicted zinc leaching efficiency of the remained 30 samples (Fig. 7). The SSR value and AIC value of MLR model (5507.5 ± 949.8; 6.54) and nnet model (5128.0 ± 1220.0; 6.47) showed no significance, which were both smaller than that of neuralnet model (9277.4 ± 2749.7; 7.06). Therefore, it indicated that in this simple system, MLR models might predict the function of zinc leaching efficiency well.

## Discussion

Temperature and energy resource components are two major factors in biological processes (Rosenblatt and Schmitz 2016), such as sewage sludge treatment (Tyagi et al. 1994; Wong et al. 2015; Pathak et al. 2009), methane generation (Conrad et al. 2009; Yvon-Durocher et al. 2014), and organic matter (aromatic, dairy manure, cellulose, etc.) degradation (Hartmann and Ahring 2005), affecting microbial community. We explored the responses of the microbial community and eco-system function (metal recovery ability) to temperature or nutrient/energy resource in bioleaching system due to its simple and relatively well-known specificity. Via converting the microbial community and physicochemical parameters, Temperature (Behrad Vakylabad 2011; Watling et al. 2016) and energy resource (Sreekrishnan et al. 1996; Garrido et al. 2008) affected mineral dissolution. Our results indicated that composition and structure of microbial community were temperature sensitive, while physicochemical parameters were mainly affected by the OMCs. Besides, temperature (accompanying variations of the microbial community) and the OMCs (accompanying variations of physicochemical parameters) could both convert and predict zinc leaching efficiency.

### Temperature altered microbial community and zinc leaching efficiency

Temperature is one of the main environmental factors governing microbial life, which is a kind of selective pressure for activity, density and composition of microorganisms (Aragno 1981). Therefore, there is no wonder that microorganisms are most often chosen as models for studying the effects of temperature on biological processes. Some species ranged from low to high abundance under temperature variation, and similar results were obtained in different fields, such as freshwater pond (Lear et al. 2014), marine (Gilbert et al. 2012), and soil (Deangelis et al. 2015; Karhu et al. 2014). Deangelis et al. (2015) reported that some members of the *Actinobacteria*, *Alphaproteobacteria* and *Acidobacteria* showed strong warming responses in temperate forest soils, with one *Actinomycete* decreasing from 4.5 to 1% relative to warming (5 °C increased). A temperature increase of 5–10 °C had little effect on the microbial community structure and an increase of 25 °C shifted the concurrent species, e.g., *Arcobacter* and *Marinobacter* (Canion et al. 2014). The temperature sensitivity of microorganisms also applied to bioleaching systems. Although both iron- and sulfur-oxidizing microbes were detected at 33, 45 and 65 °C, iron-oxidizing microbes (e.g., *L. ferriphilum*) were the dominant taxa at 33 and 45 °C and sulfur-oxidizing microbes (e.g., uncultured sulfur-oxidizing microbes symbiont bacteria) were the predominant at 65 °C in column bioleaching of chalcopyrite (Chen et al. 2014). In our study, the taxa occupied the same ecological niche (oxidizing sulfur or iron), e.g., OTU_3 (> 97 similarity to *L. ferriphilum*) and OTU_28 (> 82 similarity to *L. ferrooxidans*), and who would become the winner when they competed with each other was determined by the limiting factors (e.g., temperature) (Bowker et al. 2010; Kreimer et al. 2012). OTU_3 grew better than OTU_28 at the relatively high temperature (35–45 °C), and they showed contrast growth at low temperature (30 °C). This could be well explained by their optimal growth temperature (Gao et al. 2007; Harrison and Norris 1985). Temperature affected the microbial community, via the difference of the generation time of microorganisms, except for growth condition of microorganisms. The generation time of microorganisms could be shortened at a high-growable temperature (Plank and Harvey 1979), indicating that high temperature, to some extent, could accelerate the growth of microorganisms. Furthermore, it was reported that temperature was more important than trophic interaction in influencing microbial community (Gilbert et al. 2012), and the temperature dependence of microbial diversity is greatest at extreme nutrient level (Wang et al. 2016). Our results also showed that microbial community structure was sensitive to temperature, rather than to the OMCs (energy resource).

Temperature affects biological processes (Aragno 1981; Karhu et al. 2014; Williamson et al. 2016; Zhou et al. 2016), via changing microbial community composition, structure and diversity. Furthermore, temperature could alter the activity of enzymes (Bromfield et al. 2011; Razavi et al. 2017). In addition, temperature could alter the chemical dissolution speed of mineral (Chen et al. 2014) and the solubility of bioleaching of product (e.g., jarosite and calcium sulfate) (Sand et al. 2001). Above all, it was indicated that temperature affected sphalerite bioleaching through determining the composition and growth speed of microorganisms. These findings were consistent with our result that zinc recovery increased with increasing temperature in S and SP groups (Fig. 4a).

### Original mineral composition altered physicochemical parameters and zinc leaching efficiency

In natural ecosystem, energy resource/fertilization can convert physicochemical parameters (Karami et al. 2012), e.g., pH and [Ca]. The OMCs as substrates changed energy sources of bioleaching system, following the change of physicochemical parameters (e.g., pH and [Fe^3+^]) in leachate as well (Xiao et al. 2015). The dissolution sketch map of 4 different OMCs at 30 °C was shown in Additional file 1: Figure S4, which showed physicochemical parameters were obviously different among four groups, and PCA and PL-SPM showed that the whole of physicochemical parameters was affected by the OMCs and bioleaching stages, rather than temperature.

Energy resource, leading to changes of physicochemical parameters, is also an important factor to exert influences in microbial community (Bowen et al. 2011; Campbell et al. 2010) in natural ecosystem. In bioleaching system, *Ferroplasma* is accelerated by copper ions (Zhang et al. 2015), so that it grew well with additions of chalcopyrite (SC and SPC groups). However, due to the energy resource was sufficient, it didn’t become a limiting factor and microorganisms were more sensitive to temperature in this condition.

Energy resource/nutrient component plays an important role in the biological process. Zeglin et al. (2016) reported that quantity and source of organic matter affects microbial community structure and function following volcanic eruption. Nitrogen, one of the limiting nutrients for the yield of crops, also has an important function in methane oxidation of rice rhizosphere microbial community (Shrestha et al. 2010). Co-digestion of manure was thought as an efficient approach to enhance the digestion efficiency (Hartmann and Ahring 2005). Our previous study reported that pyrite or chalcopyrite could enhance zinc recovery at 40 °C (Xiao et al. 2015). Present study indicated that additions of pyrite always enhanced sphalerite dissolution under different temperature (30, 35, 40, 45 and 50 °C). On the one hand, sphalerite (ZnS) was polysulfide mechanism, while pyrite dissolution mechanism was thiosulfate mechanism (Fig. 7) (Sand et al. 2001). On the other hand, in contrast to sphalerite system (S group), the proportions of iron and sulfur was high in pyrite. Thus, in SP group, additional pyrite: (i) produced higher concentration of ferric iron, which could oxidized minerals as an oxidizer (Crundwell 2003); (ii) decreased pH value, which made more solubilized jarosite (Rodríguez et al. 2003a, b); and (iii) reduced the formation of elemental sulfur (Rodríguez et al. 2003a, b), which would restrict the mineral dissolving. The similar findings were reported by several previous studies (Nazari et al. 2011; Zhao et al. 2015; Lizama and Suzuki 2011) also reported that addition of pyrite enhanced sphalerite bioleaching.

However, other results in our study showed that in SC group, zinc recovery was the highest at 30 and 50 °C and the lowest at 40 °C, while in SPC group was the highest at 30 °C and was the lowest at 50 °C, which was not consistent with the regular that metal recovery increased with increasing temperature. The reasons were mainly as follows.

Firstly, the OMCs were complex. When microorganisms could utilize several energy sources in a system, it might be a question which was preferentially oxidized and temperature might affect the choice (Tsai et al. 2003). Tsai et al. (2003) reported that solubilization efficiency of total extractable Ni, Zn, Cu and Cr was higher (> 90%) at 37 °C than that at 25 and 55 °C, while Pb recovery was the highest (64.6%) at 25 °C.

Secondly, the process of sulfide dissolution was also interfered by other physicochemical parameters, other than temperature. The rate of pyrite dissolution was affected by the concentration of sulfate at temperature gradients (Basson et al. 2013). With additional low concentration of sulfate (3.94 g/L), the dissolution rate was high at high temperature (65 °C), while with additional moderate concentration of sulfate (42.1 and 78.5 g/L sulfate), it was high at 50 °C, and with additional high concentration of sulfate (119 g/L), it was high at 35 °C.

Thirdly, it might be due to the complexity of Galvanic leaching among three minerals (ZnS, FeS_2_ and CuFeS_2_) and the intermediate metabolite (e.g. CuS) (Zeng et al. 2011). Galvanic leaching was often ignored (Khmeleva et al. 2005; Lizama and Suzuki 2011), since the effects of microbes and ferric iron/pH were considered to be much more important (Xiao et al. 2015). However, although its effects are weak, it existed actually among these minerals. The electrostatic potential was ZnS < CuFeS_2_ < FeS_2_, and the mineral with higher electrostatic potential could accelerate the dissolution of the mineral with the lower (Mehta and Murr 1982). Galvanic leaching was also affected by temperature and it might be weakened at high temperature.

Besides, it was also might be that the adsorption capacity between different mineral and different microorganisms was different and the adsorption capacity of mineral for microorganisms also shifted under different temperature. Previous study found that compared to the amount of *At. ferrooxidans* R1 and SP3 attached to chalcopyrite or sphalerite, attachment to pyrite was higher, and compared to chalcopyrite, less amount of strains R1 and more amount of strains SP3 attach to sphalerite (Harneit et al. 2006). Therefore, it might explain why additional chalcopyrite increased zinc leaching efficiency at low temperature while decreased it at high temperature. This is also a common phenomenon in natural ecosystem, such that for species turnover rates (one ecosystem function), temperature effects are strongest at intermediate nutrient gradients in subtropical regions, while at two extreme ends of nutrient gradients in subarctic regions (Wang et al. 2016). That is, temperature effect on ecosystem function is versatile in ecosystem with different nutrient/energy resource condition.

### Effective models predicting zinc leaching efficiency

It was always expected to popularize the findings in experiments and provide a certain reference in the future, via constructing feasible predicting models (Venkataraman et al. 2015; Kuang et al. 2016; Pinto et al. 2014). In this experiment, we found that temperature and the OMCs, as well as the variations of microbial community and physicochemical parameters, were all correlated to zinc leaching efficiency. Taking biotic and abiotic factors, we constructed several predicting models, which were meaningful and were the first attempt (to authors’ knowledge). In contrast to model 2, model 1 and 3 could not predict sphalerite bioleaching. It suggested that ferric ions and pH occupied a vital position in sphalerite dissolution (Mousavi et al. 2008). In addition, although OTU_1 and OTU_2 were dominant OTUs, the rare OTUs and their interaction between OTUs might also play important roles in bioleaching process (Lyons and Schwartz 2001; Lyons et al. 2005). Using these five parameters, the predicting model could be optimized and was efficiently connected the biotic and abiotic effects in bioleaching process. It suggested that the cooperation of biotic and abiotic effects was inseparable and significative (Sand et al. 2001). This predicting model might also be applied in other mineral bioleaching system after modified. That was, if the iron/sulfur concentration, pH value, temperature and microorganism had been obtained in a natural bioleaching system/mine, the potential metal recovery efficiency might be estimated, which was significative for mine selection in industry applications.

## Additional file

**Additional file 1.** Supporting materials including supporting experimental methods,  figures and tables.

## Abbreviations

OMCs: original mineral compositions
S: sphalerite
SP: sphalerite adding with pyrite
SC: sphalerite adding with chalcopyrite
SPC: sphalerite adding with pyrite and chalcopyrite
MLR: multiple linear regression
AIC: Akaike information criterion
OTU: operational taxonomic unit
ORP: redox potential
PCA: principal component analysis
DCA: detrended correspondence analysis
PLS-PM: partial least squares path modeling
