# Supplementary material for: Responses of zinc recovery to temperature and mineral composition during sphalerite bioleaching process
Source: AMB Express. 2017 Oct 23;7:190. doi: 10.1186/s13568-017-0491-1 (PMC5653677; doi:10.1186/s13568-017-0491-1)
Supplement: Supplementary file 1 — Additional file 1. Supporting materials including supporting experimental methods, figures and tables. [file 13568_2017_491_MOESM1_ESM.pdf]

AMB Express

Additional file 1

Responses of zinc recovery to temperature and mineral composition during sphalerite bioleaching process

Yunhua Xiao[1,2], Xueduan Liu[2], Jun Fang[1], Yili Liang[2], Xian Zhang[2], Delong Meng[2,*], Huaqun Yin[2,*]

[1]College of Bioscience and Biotechnology and College of Agronomy, Hunan Agricultural University, Changsha 410128, China; [2]School of Minerals Processing and Bioengineering, Central South University, Changsha 410083, China

Corresponding author:

Huaqun Yin (yinhuaqun_cs@sina.com); Delong Meng (meng.delong@ucdconnect.ie)

Tel: +86(731)88830546

Fax: +86(731)88830546

Supplementary Materials and Methods

Bacterial cultures

The bacterial cultures were enriched as our previous study. Microbes from acid mine drainage in different local, including DeXing copper mine (Jiangxi, China), ZiJinShan copper mine (Fujian, China), and Chambishi copper mine (Zambia), were mixed. The mixed cultures were incubated in 9K medium with different energy source ($FeSO_4$, S^0, $FeSO_4$ + S^0 + 0.01% wt/vol yeast extraction), different temperature (30 ℃, 40 ℃, 50 ℃) and different pH (1.0, 1.8, 2.5), respectively. And then the incubated cultures were mixed together as the bacterial cultures in the next shake flask bioleaching tests.

Minerals

Three minerals, including sphalerite, pyrite and chalcopyrite, were utilized in this study. They were prepared by grinding and sieving to make sure particle diameter below 75 μm. The elemental composition and mineral composition were measured by Inductively Coupled Plasma-Atomic Emission Spectrometry (ICP-AES) and X-ray diffraction (XRD), respectively.

Bioleaching tests

Bioleaching tests were carried out in 250 mL shake flasks with 100 mL 9K medium. The flasks were sterilized by autoclaving for 25 min at 121 ℃ and 101Kpa. On the other hand, the mineral were sterilized by autoclaving for 40 min at 110 ℃ and 101KPa. The initial pH value, pulp density and cells density were 2.0, 2% wt/vol and 5×10^6 cells/mL, respectively in this experiment. During the experiment, distilled water and 9K medium were added periodically to the flasks to compensate for the evaporation loss and sampling loss. There were four treatments with different mineral composition, which were sphalerite only (S), added with pyrite (SP, w/w, 1: 1), with chalcopyrite (SC, w/w, 1:1) and with both (SPC, w/w/w, 1:1:1). This experiment was conducted at 170 r/min and at 30, 35, 40, 45 and 50℃, respectively. All treatments (4 mineral treatments×5 temperature treatments) were carried out in septuplicate.

The physicochemical parameters, including pH, redox potential (ORP), the concentration of dissolved ferrous ion, total iron, copper ion, zinc ion and sulfate ion in the solution were monitored and a flask of each experimental group was removed for DNA extraction on day 6, 12, 21 and 30.

Detection techniques of physicochemical parameters

The pH value and ORP were measured using pH meter and potentiometer, respectively. The dissolved ferrous ion, copper ion and sulfate ion were measured using ultraviolet spectrophotometry with microplate reader (ELIASA) by Atomic Absorption Spectrophotomety (AAS). Zinc ion of leaching solution was measured by ICP-AES. Zinc leaching efficiency (ZLE) was calculated by using the formula ZLE = $[Zn^{2+}]$*100 mL/T_{Zn}, where $[Zn^{2+}]$ is the concentration of zinc ions in each treatment, T_{Zn} is the total zinc in each treatment.

Collection of microorganisms, nucleic acid extraction and quantification

Firstly, to separate supernatant from the mineral residue, the cells of solution were filtered out using filter paper (15~20 μm). Secondly, the residue was washed with sterilized distilled water, vortex oscillating vigorously using oscillator for 1 min to transfer cells from mineral surface to solution. 30 ml was used once and repeated for several times until few cells (low than 10^5 cells/mL) could be counted in elution buffer. Thirdly, mix up the elution buffer with the leaching solution and centrifuged at 13,400 g for 18 min to collect the cells in centrifuge bottle. Finally, total DNA was extracted from each treatment using a TIANamp genomic DNA purification kit (Tiangen Biotech, Co., Ltd., Beijing, China). 0.8% (w/v) agarose gel was used to visualize the extracted DNA stained with ethidium bromide, while

a NanoDrop_ND-1000 spectrophotometer (NanoDrop Technologies, Wilmington, USA) was used to measure the concentration of purified total genomic DNA.

DNA amplification, Illumina sequencing and data processing for DNA samples

Tagged PCR products were generated using primer pairs with unique barcodes through PCR. The V4 region of the 16S rRNA genes was amplified with primer pair. The primer pair was 515F (5'-GTGCCAGCMGCCGCGGTAA-3') and 806R (5'-GGACTACHVGGGTWTCTAAT-3') combined with Illumina adapter sequences, a pad and a linker of two bases, and barcodes on the reverse primers. The 50 μl 16s rRNA amplification reaction mixtures included 0.5 μL of Taq DNA polymerase (TaKaRa), 5 μL of 10×PCR buffer, 1.5 μL of dNTP Mix, 1.5 μL of forward primers(10 μM) , 1.5 μL of reverse primers (10 μM), 1 μL of DNA extracts(20~30 ng/μL) and 39 μL of ddH_2O. Samples were amplified using the following program: denaturation at 94 ℃ for 1min, and 30 cycles of 94 ℃ for 20 s, 53 ℃ for 25 s, and 68 ℃ for 45 s, with a final extension at 68 ℃ for 10min. Positive PCR products were confirmed by agarose gel electrophoresis and recovered according to the instruction of DNA gel extraction kit. The concentration of PCR products was quantified with a NanoDrop ND-1000 Spectrophotometer (NanoDrop Technologies, Wilmington, USA). Illumina 2x250 bp paired-ends sequencing was performed on Miseq machine (Illumina, San Diego, CA) by using Miseq 500 cycles kit (Wu et al., 2015).

The raw data of samples for Miseq paired-end sequencing was FASTQ data format, and then the adapters and other Illumina-specific sequences from the read were cut and removed. Sequences perfect matching to barcodes were split into sample libraries and then were trimmed by using Btrim. The quantity of reads joined together was counted by forward and reverse reads with at least 10 bp overlap and lower than 5% mismatches through Flash (Edgar, 2010). The joined pairs were then handled with many steps, such as removal of sequences less than 220 bp with ambiguous base 'N' and an average base quality score lower than 20. The trimmed sequences were chimera detected and removed using the Uchime algorithm (Edgar et al., 2011). The remaining and unique sequences were clustered at 97% similarity level to generate operational taxonomic units (OTUs) by UCLUST, and taxonomic assignment was conducted by RDP classifier with a minimal 50% confidence degree. The above-mentioned steps were conducted through the in-lab Galaxy pipeline platform.

References

Edgar, R.C. (2010) Search and clustering orders of magnitude faster than BLAST. *Bioinformatics* **26**: 2460-2461.

Edgar, R.C., Haas, B.J., Clemente, J.C., Quince, C., and Knight, R. (2011) UCHIME improves sensitivity and speed of chimera detection. *Bioinformatics* **27**: 2194-2200.

Wu, L., Wen, C., Qin, Y., Yin, H., Tu, Q., and Nostrand, J.D.V. et al. (2015) Phasing amplicon sequencing on Illumina Miseq for robust environmental microbial community analysis. *BMC Microbiol* **15**: 1-12.

Yuguang, W., Weimin, Z., Guanzhou, Q., Xinhua, C., and Hongbo, Z. (2014) A moderately thermophilic mixed microbial culture for bioleaching of chalcopyrite concentrate at high pulp density. *Applied & Environmental Microbiology* **80**: 741-750.

Supplementary Figures

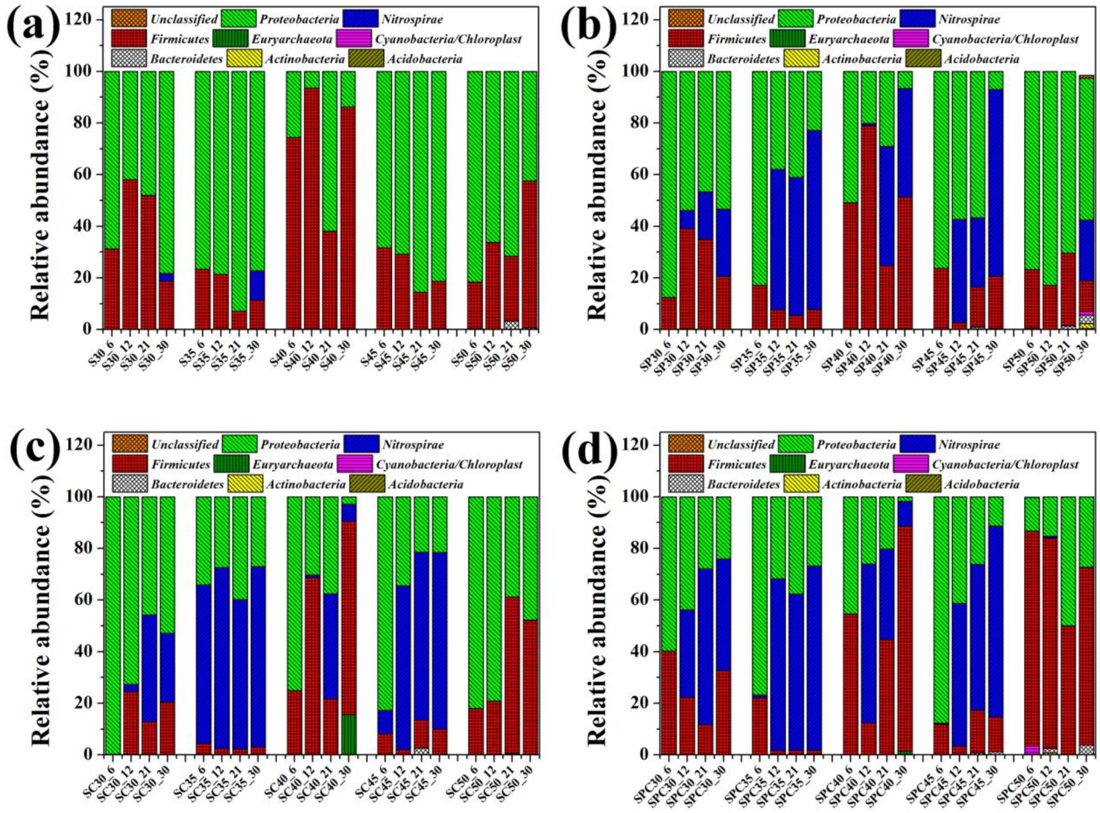

Figure S1 The compositions of microbial community at phylum level

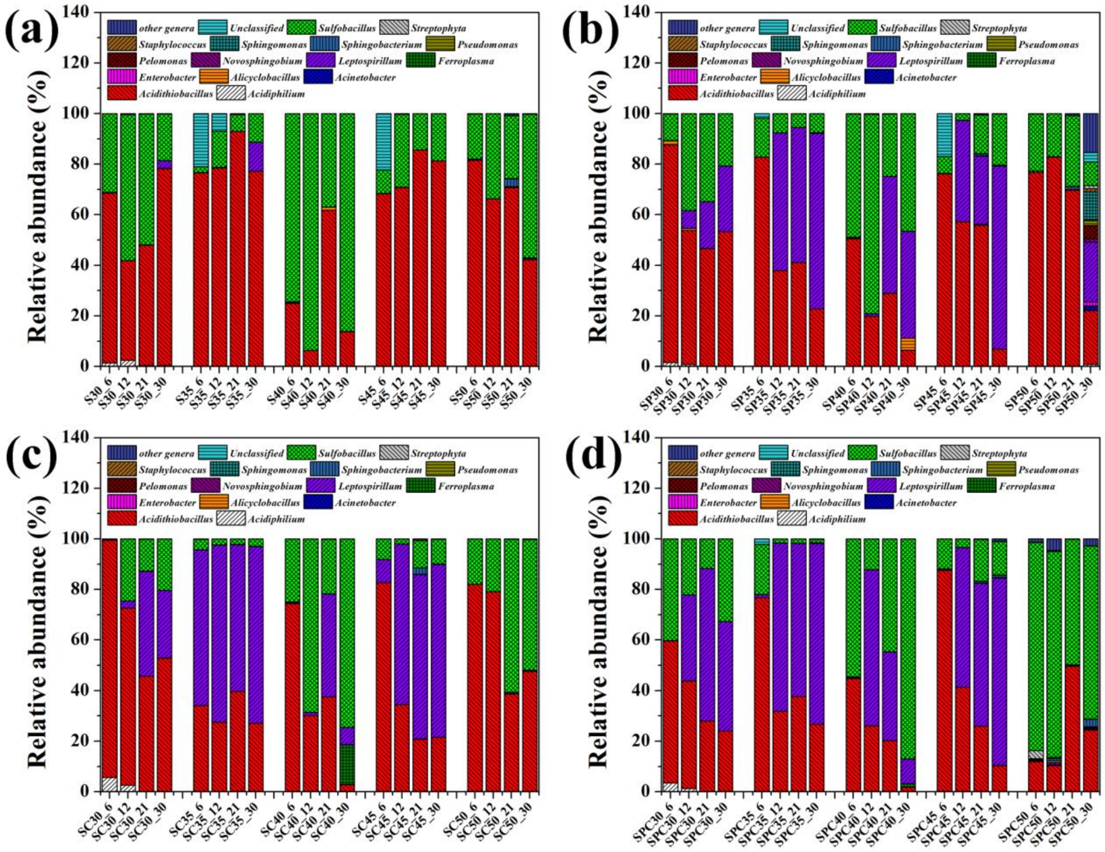

Figure S2 The compositions of microbial community at genus level

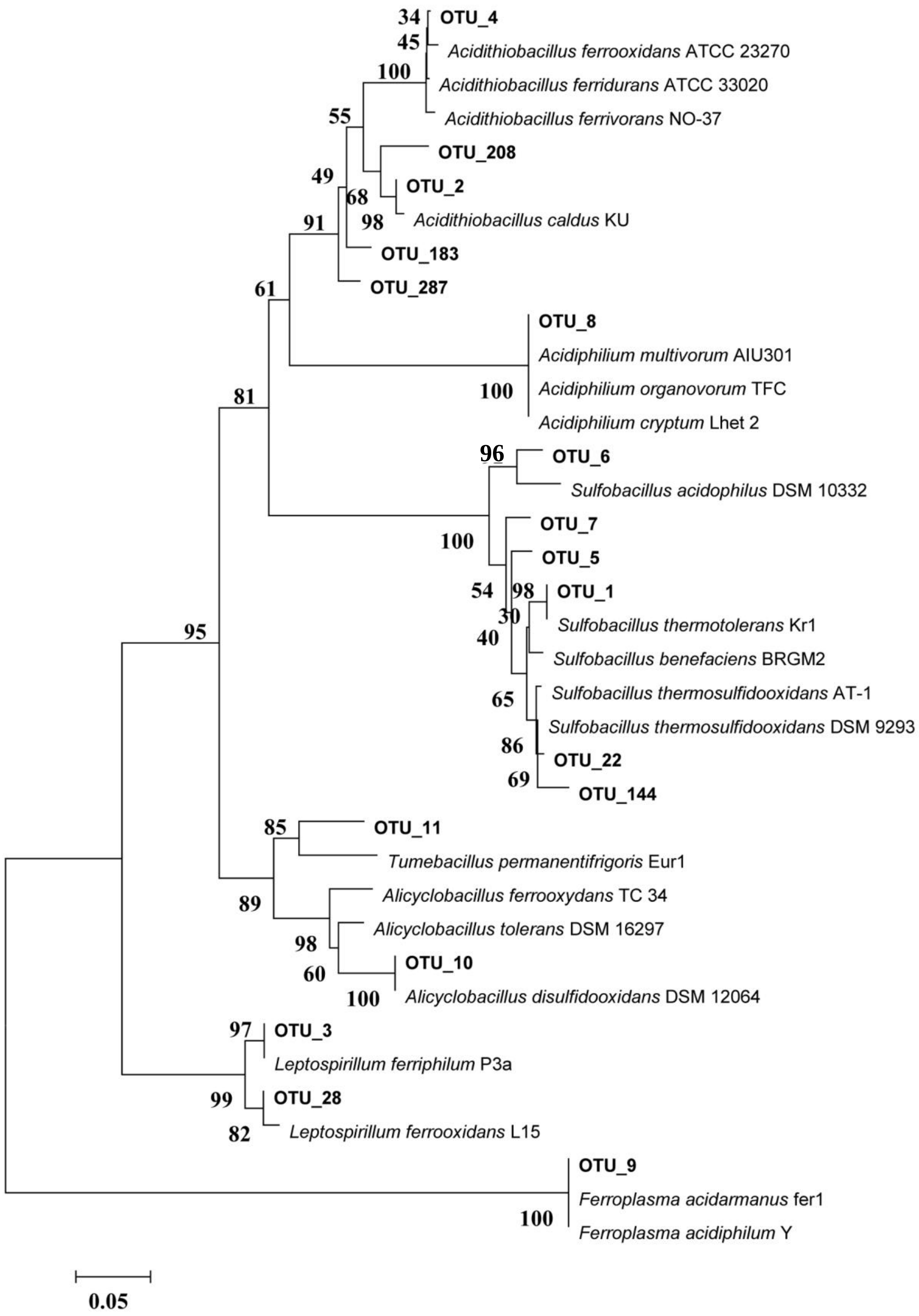

Figure S3 Maximum likelihood trees based on partial 16S rRNA gene sequences from the seventheen OTUs. The sequences obtained in this study are indicated in bold.

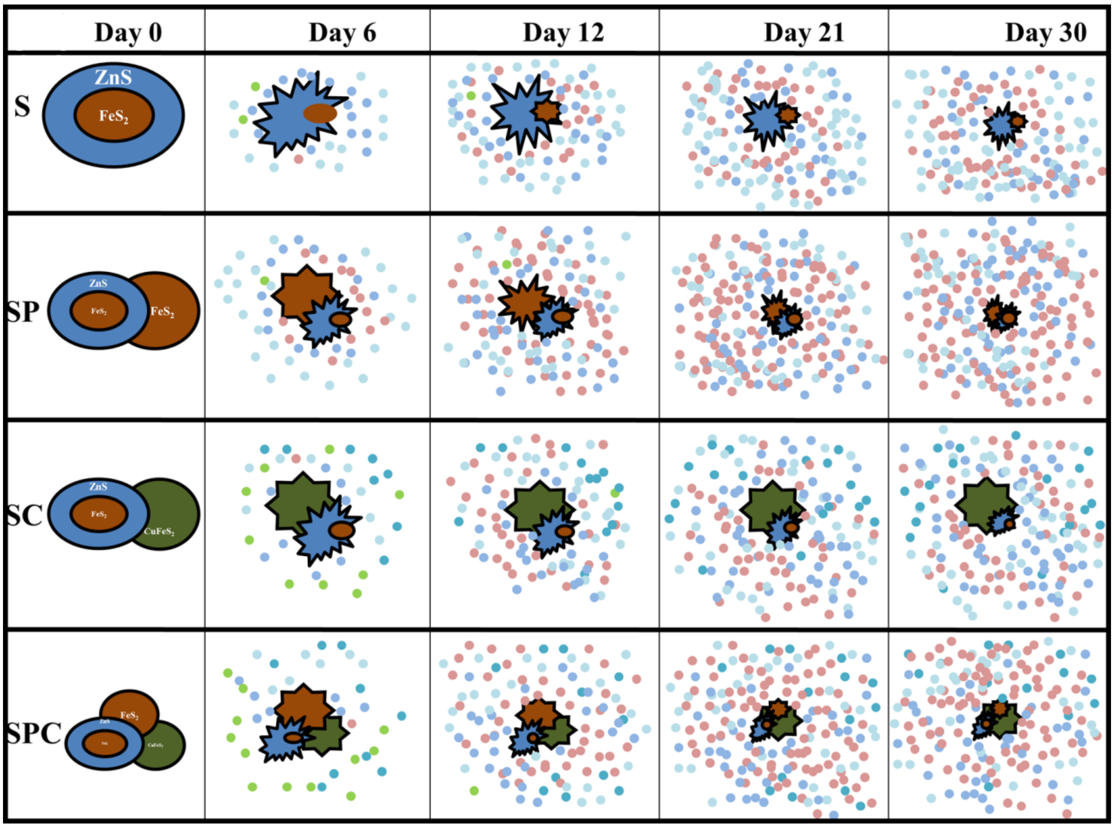

Figure S4 The dissolution sketch map of four different mineral composition groups at 30℃

Supplementary Tables

Table S1 Element and mineral compositions of the three minerals

Element/ Mineral (%)	Sphalerite	Pyrite	Chalcopyrite
S	35.190	50.020	25.730
P	0.066	0.140	0.170
Fe	23.570	41.62	29.59
Ca	0.940	0.680	1.090
Na	0.059	0.039	0.053
Al	0.080	0.180	0.100
Zn	34.170	0.050	0.260
Mg	0.021	0.190	0.300
Mn	0.04	0.053	0.008
K	0	0	0.062
Pb	2.710	0.003	0.027
Cu	0.024	0.096	30.74
Ni	0	0	0.010
Ag	0.004	0	0.004
Co	0	0	0.005
Cd	0.079	0	0
As	0	0.110	0
ZnS	66.84	-	-
FeS_2	26.75	99.00	3.85
$CuFeS_2$	-	-	90.89

Table S2 Zinc leaching efficiency at different stages (Mean ±SD, unit: %).

Sample	Day 6	Day 12	Day 21	Day 30
S30	12.5 ±0.6	33.9 ±1.7	57.7 ±2.9	66.3 ±3.3
S35	17.1 ±0.9	32.4 ±1.6	57.5 ±2.9	72.2 ±3.6
S40	13.5 ±0.7	27.8 ±1.4	63.6 ±3.2	71.2 ±3.6
S45	46.1 ±2.3	72.0 ±3.6	78.3 ±3.9	78.2 ±3.9
S50	37.3 ±1.9	81.8 ±4.1	93.6 ±4.7	89.1 ±4.5
SP30	47.5 ±2.4	57.2 ±2.9	79.6 ±4.0	80.7 ±4.0
SP35	45.3 ±2.3	74.5 ±3.7	90.2 ±4.5	86.6 ±4.3
SP40	48.7 ±2.4	54.2 ±2.7	85.2 ±4.3	84.6 ±4.2
SP45	66.4 ±3.3	89.4 ±4.5	97.6 ±5.1	94.5 ±4.7
SP50	59.9 ±3.0	93.9 ±4.7	99.99 ±5.2	97.2 ±4.9
SC30	18.1 ±0.9	51.0 ±2.5	77.8 ±3.9	84.4 ±4.2
SC35	43.6 ±2.2	67.3 ±3.4	78.0 ±3.9	77.9 ±3.9
SC40	24.0 ±1.2	44.8 ±2.2	72.4 ±3.6	74.1 ±3.7
SC45	34.4 ±1.7	70.3 ±3.5	79.4 ±4.0	77.9 ±3.9
SC50	36.8 ±1.8	53.8 ±2.8	66.7 ±3.3	82.9 ±4.1
SPC30	23.5 ±1.2	60.0 ±3.0	88.3 ±4.4	85.8 ±4.3
SPC35	28.2 ±1.4	70.6 ±2.0	81.2 ±4.1	78.7 ±3.9
SPC40	25.7 ±1.3	56.7 ± 3.5	80.2 ±4.0	84.4 ±4.2
SPC45	38.5 ±1.9	59.6 ±2.7	87.5 ±4.4	81.9 ±4.1
SPC50	39.4 ±2.0	39.4 ±3.0	36.8 ±1.8	78.9 ±3.9

Table S3(a) Physicochemical parameters

Sample	pH	ORP/mV	Fe^{2+}/ppm	Fe^{3+}/ppm	SO_4^{2-}/ppm	Zn/ppm	Cu/ppm
S30_6	1.717	373.333	103.799	26.417	2128.410	855.210	3.030
S30_12	1.553	560.000	40.345	918.850	3051.910	2315.520	5.880
S30_21	1.367	592.333	20.731	1938.523	5412.560	3944.120	7.200
S30_30	1.300	645.000	19.607	2566.899	6745.900	4529.260	4.940
S35_6	2.520	283.500	162.361	9.194	1907.210	1232.420	2.413
S35_12	1.645	413.500	13.478	265.343	3051.390	2456.880	3.577
S35_21	1.700	575.000	11.000	965.325	4470.860	4763.990	6.943
S35_30	1.520	614.500	2.000	2399.628	6647.590	6575.890	5.770
S40_6	1.627	360.000	112.849	30.648	2483.600	921.650	4.000
S40_12	1.497	543.667	34.012	680.004	2893.440	1898.440	6.000
S40_21	1.307	546.000	22.366	1912.371	5907.100	4343.230	8.000
S40_30	1.215	553.000	32.479	2535.639	6663.930	4865.410	5.000
S45_6	1.800	381.000	171.555	36.777	3315.170	3351.460	4.257
S45_12	1.595	490.000	26.963	475.585	4643.420	5588.660	5.733
S45_21	1.550	515.500	12.000	1566.016	5988.160	6777.140	6.273
S45_30	1.315	532.000	2.000	1893.944	7669.650	7635.790	6.900
S50_6	2.440	338.500	150.102	30.648	2918.060	2768.780	3.521
S50_12	1.485	468.000	17.462	687.359	5966.460	6658.100	6.253
S50_21	1.215	608.500	13.000	2365.916	9152.460	8881.140	7.296
S50_30	0.860	670.500	2.000	3778.766	13145.700	10147.100	7.318
SP30_6	1.683	569.333	35.209	681.396	3021.850	1624.830	12.080
SP30_12	1.297	612.000	42.797	2879.881	4472.670	1957.640	12.270
SP30_21	1.057	700.667	24.613	5040.255	8142.070	2724.160	14.920
SP30_30	1.035	708.500	33.092	5372.984	9226.770	2762.740	15.080
SP35_6	2.080	340.500	588.361	12.351	2743.310	1633.470	11.264
SP35_12	1.625	550.500	8.268	1148.604	4549.940	2832.170	11.584
SP35_21	1.280	693.500	2.000	4048.464	7215.540	3740.440	12.022
SP35_30	1.105	690.000	2.000	5372.436	8663.400	3949.040	11.905
SP40_6	1.643	493.667	81.180	510.792	3027.320	1666.290	13.000
SP40_12	1.337	549.667	44.432	1922.045	3639.340	1853.880	13.000
SP40_21	0.897	697.333	23.183	4167.210	8204.910	2913.960	14.000
SP40_30	0.815	712.500	27.269	4710.692	9387.970	2895.780	15.000
SP45_6	2.785	362.500	499.483	49.036	3244.430	2416.910	11.899
SP45_12	1.545	494.500	6.429	1179.865	4498.160	3477.490	12.525
SP45_21	1.015	699.500	2.000	4296.708	8945.130	4404.190	12.898
SP45_30	0.845	700.000	2.000	5832.148	11070.800	4619.350	13.056
SP50_6	2.995	350.500	487.224	15.324	2852.460	2227.890	10.409
SP50_12	1.400	488.000	36.463	717.087	4873.170	3823.670	11.801
SP50_21	1.025	588.500	2.000	2963.542	8331.390	4940.870	12.859
SP50_30	0.795	650.000	2.000	6248.954	12394.200	5546.070	13.567

Table S3(b) Physicochemical parameters

Sample	pH	ORP/mV	Fe^{2+}/ppm	Fe^{3+}/ppm	SO_4^{2-}/ppm	Zn/ppm	Cu/ppm
SC30_6	1.830	347.000	468.871	20.000	2729.500	824.830	402.000
SC30_12	1.540	600.667	39.732	1616.183	3830.600	1541.900	688.000
SC30_21	1.083	714.000	18.483	2451.562	6540.980	2492.680	926.000
SC30_30	1.025	706.500	14.704	2069.184	6407.100	2552.540	1005.000
SC35_6	1.630	352.000	444.353	600.180	2661.200	623.500	397.070
SC35_12	1.563	611.000	38.098	1630.076	3704.910	1755.210	666.400
SC35_21	1.343	705.333	19.301	2724.530	6005.460	2679.600	874.510
SC35_30	1.285	702.000	20.527	2780.512	6672.130	2907.310	989.820
SC40_6	2.165	427.500	183.814	297.281	3383.870	1581.340	677.072
SC40_12	1.620	585.500	9.800	1021.417	4319.150	2574.550	954.409
SC40_21	1.415	685.000	2.000	1566.016	5363.230	3255.820	1025.160
SC40_30	1.270	685.500	2.000	1995.081	6110.310	3576.940	1127.300
SC45_6	1.870	365.000	401.411	42.907	3242.160	1259.220	996.141
SC45_12	1.620	611.500	21.446	891.472	4697.230	2752.150	1246.520
SC45_21	1.250	685.000	2.000	1789.743	6029.270	3461.500	1358.450
SC45_30	1.145	682.500	2.000	2273.973	6985.280	3831.250	1491.590
SC50_6	2.580	360.500	404.476	15.324	2882.860	1375.480	688.370
SC50_12	2.110	420.500	18.381	1091.293	3904.990	2206.190	1479.970
SC50_21	1.480	526.000	2.000	1538.433	5824.410	3188.070	2034.970
SC50_30	1.155	669.500	2.000	2604.966	8575.370	4754.610	2675.000
SPC30_6	1.857	378.333	595.037	49.036	2590.160	540.790	482.860
SPC30_12	1.430	638.667	40.141	2134.739	4068.300	1380.040	555.920
SPC30_21	1.177	720.667	21.548	4062.599	7281.420	2029.040	707.090
SPC30_30	1.140	713.500	26.043	4098.968	7726.770	1971.610	709.390
SPC35_6	2.240	347.000	487.224	27.583	2609.790	681.645	491.966
SPC35_12	1.710	576.500	7.348	1246.063	4354.510	1802.980	612.646
SPC35_21	1.315	690.000	2.000	2516.089	5501.270	2261.980	643.327
SPC35_30	1.160	691.500	2.000	3193.398	6346.400	2411.770	682.599
SPC40_6	1.673	365.333	611.382	96.029	2647.540	591.350	493.000
SPC40_12	1.330	646.667	39.528	2259.985	4210.380	1302.210	603.000
SPC40_21	0.940	728.000	21.140	3887.296	7500.000	1842.790	723.000
SPC40_30	0.885	725.500	22.979	3869.112	7920.760	1939.020	733.000
SPC45_6	2.230	372.500	569.972	64.360	2594.020	940.820	698.955
SPC45_12	1.600	514.000	68.950	666.519	3844.200	1557.050	787.134
SPC45_21	1.175	693.000	2.000	2721.427	6770.560	2547.020	1032.740
SPC45_30	1.025	693.000	2.000	4345.744	8165.330	2689.630	1098.880
SPC50_6	2.580	360.000	646.591	39.842	2244.910	985.013	631.734
SPC50_12	2.720	403.500	23.591	230.712	2368.640	1078.280	1048.060
SPC50_21	1.540	515.500	2.000	563.843	3363.260	1175.180	1338.330
SPC50_30	1.100	642.000	2.000	2822.564	7884.330	3024.260	1688.950

Table S4 Shannon diversity index at different stages

Sample	Day 6	Day 12	Day 21	Day 30
S30	1.441	1.239	1.257	1.480
S35	0.812	1.020	0.865	1.323
S40	0.648	0.301	0.778	0.479
S45	0.949	0.876	0.474	0.545
S50	0.641	0.753	0.849	0.849
SP30	1.355	1.566	2.136	2.020
SP35	1.456	1.381	1.307	1.032
SP40	1.014	0.800	1.406	1.509
SP45	0.748	0.822	1.176	0.823
SP50	0.639	0.543	0.835	3.253
SC30	0.620	1.353	1.807	1.795
SC35	0.937	0.950	1.138	0.935
SC40	0.871	1.160	1.333	1.555
SC45	0.677	0.768	1.086	0.932
SC50	0.621	0.639	0.845	0.922
SPC30	1.492	1.874	1.312	1.764
SPC35	1.472	1.010	1.076	0.881
SPC40	1.227	1.055	1.556	1.726
SPC45	0.532	0.869	1.146	0.961
SPC50	0.804	0.902	0.910	1.086

Table S5 The correlation between zinc leaching efficiency on day 30 and OTU at different stages

OTU	Day 6		Day 12		Day 21		Day 30	
	r	p	r	p	r	p	r	p
OTU_1	-0.384	0.095	-0.435	0.055	**-0.538**	**0.014**	-0.304	0.192
OTU_2	0.380	0.098	**0.457**	**0.043**	0.197	0.405	-0.217	0.359
OTU_3	-0.101	0.671	0.230	0.329	0.091	0.704	0.357	0.122
OTU_4	-0.071	0.768	-0.177	0.456	**-0.543**	**0.014**	-0.337	0.147
OTU_5	**0.509**	**0.022**	0.184	0.437	0.277	0.237	-0.026	0.915
OTU_6	0.000	1.000	0.110	0.644	-0.438	0.053	0.171	0.471
OTU_7	0.207	0.381	0.215	0.362	0.128	0.591	0.075	0.753
OTU_8	0.220	0.352	-0.024	0.920	-0.337	0.146	0.312	0.180
OTU_9	0.363	0.116	0.320	0.169	-0.136	0.568	-0.141	0.555
OTU_10	0.070	0.768	0.152	0.523	-0.263	0.263	0.120	0.613
OTU_11	-0.217	0.359	-0.130	0.584	-0.327	0.159	0.000	1.000
OTU_12	0.000	1.000	0.000	1.000	0.000	1.000	0.321	0.168
OTU_13	0.026	0.913	-0.139	0.559	0.300	0.199	**0.509**	**0.022**
OTU_14	-0.053	0.825	-0.258	0.272	0.000	1.000	-0.394	0.085
OTU_15	-0.219	0.355	0.000	1.000	**0.568**	**0.009**	0.418	0.067
OTU_16	0.102	0.670	-0.080	0.739	0.177	0.454	-0.073	0.761
OTU_17	-0.023	0.924	-0.312	0.181	-0.420	0.065	0.000	1.000
OTU_18	0.000	1.000	0.147	0.536	-0.079	0.742	0.230	0.329
OTU_19	0.000	1.000	0.000	1.000	0.051	0.833	0.034	0.888
OTU_20	-0.057	0.811	0.106	0.657	-0.182	0.444	-0.391	0.088
OTU_21	0.297	0.204	0.000	1.000	-0.098	0.680	0.393	0.087
OTU_22	0.164	0.489	0.070	0.769	0.261	0.267	0.153	0.519
OTU_23	0.395	0.085	0.179	0.451	0.000	1.000	0.000	1.000
OTU_24	0.130	0.586	-0.002	0.995	0.272	0.247	0.000	1.000
OTU_25	0.165	0.486	0.000	1.000	0.000	1.000	0.000	1.000
OTU_26	-0.107	0.654	-0.214	0.365	0.044	0.855	**0.469**	**0.037**
OTU_27	0.000	1.000	-0.011	0.963	0.091	0.702	0.373	0.106
OTU_28	-0.002	0.992	0.239	0.311	0.164	0.490	0.001	0.997
OTU_29	0.106	0.657	**-0.562**	**0.010**	0.328	0.158	0.406	0.076
OTU_30	0.058	0.807	-0.042	0.859	0.271	0.248	0.418	0.066
OTU_31	0.000	1.000	0.000	1.000	0.097	0.686	**0.450**	**0.047**
OTU_32	0.129	0.588	0.000	1.000	0.346	0.135	-0.130	0.585
OTU_33	0.000	1.000	0.000	1.000	0.000	1.000	0.419	0.066
OTU_34	0.000	1.000	0.000	1.000	0.000	1.000	0.000	1.000
OTU_35	0.000	1.000	0.104	0.664	-0.048	0.841	0.417	0.067
OTU_36	0.000	1.000	0.000	1.000	0.000	1.000	0.403	0.078
OTU_37	0.000	1.000	0.119	0.617	0.000	1.000	-0.312	0.181
OTU_38	0.312	0.181	-0.029	0.904	-0.162	0.495	0.247	0.294
OTU_39	-0.219	0.355	0.000	1.000	0.404	0.077	0.113	0.635
OTU_40	0.060	0.802	0.000	1.000	0.127	0.595	**0.517**	**0.020**

OTU_41	0.309	0.185	-0.068	0.777	0.412	0.071	0.312	0.181
OTU_42	0.000	1.000	-0.335	0.149	0.084	0.724	-0.252	0.284
OTU_43	0.000	1.000	0.000	1.000	0.000	1.000	0.000	1.000
OTU_44	0.000	1.000	0.000	1.000	0.074	0.757	0.220	0.352
OTU_45	0.000	1.000	0.000	1.000	0.081	0.735	0.010	0.967
OTU_46	0.273	0.245	-0.122	0.607	0.257	0.275	0.386	0.093
OTU_47	0.006	0.979	0.000	1.000	0.000	1.000	0.000	1.000
OTU_48	-0.021	0.931	0.000	1.000	0.353	0.127	**0.496**	**0.026**
OTU_49	0.000	1.000	0.000	1.000	0.000	1.000	0.330	0.155
OTU_50	0.000	1.000	0.000	1.000	0.000	1.000	0.376	0.102
OTU_51	0.000	1.000	0.000	1.000	**0.674**	**0.001**	**0.494**	**0.027**
OTU_52	0.000	1.000	0.000	1.000	0.000	1.000	0.000	1.000
OTU_53	0.000	1.000	0.000	1.000	0.000	1.000	0.000	1.000
OTU_54	0.000	1.000	0.000	1.000	0.000	1.000	0.000	1.000
OTU_55	0.000	1.000	0.000	1.000	0.000	1.000	**0.456**	**0.043**
OTU_56	0.124	0.603	0.000	1.000	0.000	1.000	0.369	0.109
OTU_57	0.000	1.000	0.000	1.000	0.000	1.000	0.422	0.064
OTU_58	0.000	1.000	0.000	1.000	0.000	1.000	0.000	1.000
OTU_59	-0.041	0.864	0.106	0.657	-0.356	0.124	0.000	1.000
OTU_60	-0.115	0.629	0.249	0.290	0.312	0.181	0.099	0.679
OTU_61	0.000	1.000	0.000	1.000	0.000	1.000	0.400	0.081
OTU_62	0.000	1.000	0.000	1.000	0.327	0.160	-0.292	0.211
OTU_63	0.000	1.000	0.108	0.649	0.167	0.481	0.000	1.000
OTU_64	0.000	1.000	-0.042	0.859	0.141	0.553	0.230	0.329
OTU_65	0.000	1.000	0.251	0.286	0.000	1.000	0.272	0.247
OTU_66	0.000	1.000	0.000	1.000	0.000	1.000	0.356	0.124
OTU_67	0.000	1.000	0.000	1.000	0.000	1.000	0.394	0.085
OTU_68	0.000	1.000	0.000	1.000	0.000	1.000	0.000	1.000
OTU_69	0.000	1.000	0.000	1.000	0.000	1.000	0.333	0.151
OTU_70	0.000	1.000	0.000	1.000	0.000	1.000	0.000	1.000
OTU_71	0.000	1.000	0.000	1.000	-0.096	0.688	**0.489**	**0.029**
OTU_72	0.000	1.000	0.183	0.439	-0.061	0.798	**0.594**	**0.006**
OTU_73	0.167	0.482	0.000	1.000	0.000	1.000	0.416	0.068
OTU_74	0.000	1.000	0.000	1.000	0.000	1.000	0.000	1.000
OTU_75	0.220	0.352	0.000	1.000	0.012	0.959	**0.573**	**0.008**
OTU_76	0.000	1.000	0.000	1.000	0.000	1.000	0.000	1.000
OTU_77	0.000	1.000	0.000	1.000	0.000	1.000	0.386	0.093
OTU_78	0.285	0.223	0.000	1.000	0.000	1.000	0.276	0.239
OTU_79	0.000	1.000	0.000	1.000	0.000	1.000	0.310	0.184
OTU_80	0.000	1.000	0.000	1.000	0.000	1.000	0.000	1.000
OTU_81	0.000	1.000	0.000	1.000	0.000	1.000	0.400	0.081
OTU_82	0.000	1.000	0.000	1.000	0.000	1.000	0.000	1.000
OTU_83	0.000	1.000	0.000	1.000	0.000	1.000	0.000	1.000

OTU_84	0.000	1.000	0.000	1.000	0.000	1.000	0.000	1.000
OTU_85	0.000	1.000	0.000	1.000	0.000	1.000	0.000	1.000
OTU_86	0.000	1.000	0.000	1.000	0.000	1.000	-0.084	0.724
OTU_87	0.000	1.000	0.000	1.000	0.142	0.551	0.000	1.000
OTU_88	0.000	1.000	0.000	1.000	-0.308	0.186	0.000	1.000
OTU_89	0.000	1.000	0.000	1.000	0.000	1.000	0.000	1.000
OTU_90	0.000	1.000	0.000	1.000	0.000	1.000	0.000	1.000
OTU_91	0.000	1.000	0.000	1.000	0.000	1.000	0.000	1.000
OTU_92	0.395	0.085	0.000	1.000	0.029	0.904	0.391	0.088
OTU_93	0.000	1.000	0.000	1.000	0.000	1.000	0.000	1.000
OTU_94	0.278	0.235	0.000	1.000	-0.280	0.231	-0.312	0.181
OTU_95	0.289	0.216	0.000	1.000	0.000	1.000	0.000	1.000
OTU_96	0.000	1.000	0.000	1.000	0.000	1.000	0.000	1.000
OTU_97	0.163	0.493	0.000	1.000	0.409	0.073	0.000	1.000
OTU_98	0.000	1.000	0.000	1.000	0.000	1.000	0.000	1.000
OTU_99	0.000	1.000	0.000	1.000	0.000	1.000	0.000	1.000
OTU_100	0.000	1.000	0.000	1.000	0.331	0.155	-0.064	0.789
OTU_101	0.000	1.000	0.000	1.000	0.000	1.000	0.000	1.000
OTU_102	0.134	0.575	0.000	1.000	0.000	1.000	0.000	1.000
OTU_103	0.000	1.000	0.000	1.000	0.000	1.000	0.417	0.067
OTU_104	-0.390	0.090	-0.081	0.733	-0.326	0.161	0.420	0.065
OTU_105	0.342	0.140	0.262	0.264	0.000	1.000	0.000	1.000
OTU_106	0.000	1.000	0.000	1.000	0.000	1.000	0.000	1.000
OTU_107	0.000	1.000	0.000	1.000	0.000	1.000	0.000	1.000
OTU_108	0.000	1.000	0.000	1.000	-0.141	0.553	**0.498**	**0.026**
OTU_109	0.023	0.925	0.000	1.000	-0.203	0.391	0.000	1.000
OTU_110	0.000	1.000	0.000	1.000	0.000	1.000	0.000	1.000
OTU_111	0.000	1.000	0.321	0.168	0.000	1.000	0.420	0.065
OTU_112	0.000	1.000	0.000	1.000	0.000	1.000	0.000	1.000
OTU_113	-0.335	0.149	0.000	1.000	0.000	1.000	0.158	0.506
OTU_114	0.000	1.000	0.000	1.000	0.000	1.000	0.000	1.000
OTU_115	0.000	1.000	0.000	1.000	0.000	1.000	0.000	1.000
OTU_116	0.000	1.000	0.000	1.000	-0.141	0.553	0.394	0.085
OTU_117	0.000	1.000	0.000	1.000	0.000	1.000	0.000	1.000
OTU_118	-0.173	0.466	-0.117	0.624	0.322	0.166	**0.532**	**0.016**
OTU_119	0.000	1.000	0.000	1.000	0.000	1.000	0.000	1.000
OTU_120	0.000	1.000	0.000	1.000	-0.395	0.085	0.000	1.000
OTU_121	0.000	1.000	0.000	1.000	0.000	1.000	0.000	1.000
OTU_122	0.000	1.000	0.000	1.000	0.000	1.000	0.000	1.000
OTU_123	0.002	0.994	0.000	1.000	0.000	1.000	0.000	1.000
OTU_124	**0.540**	**0.014**	0.000	1.000	0.000	1.000	0.000	1.000
OTU_125	0.006	0.981	0.206	0.385	-0.285	0.223	0.247	0.294
OTU_126	0.000	1.000	0.000	1.000	0.000	1.000	0.394	0.085

OTU_127	0.000	1.000	0.000	1.000	0.000	1.000	0.000	1.000
OTU_128	0.000	1.000	0.000	1.000	0.000	1.000	0.000	1.000
OTU_129	0.000	1.000	0.000	1.000	0.000	1.000	0.000	1.000
OTU_130	0.000	1.000	0.000	1.000	0.000	1.000	0.000	1.000
OTU_131	0.000	1.000	0.000	1.000	0.000	1.000	0.000	1.000
OTU_132	0.000	1.000	0.000	1.000	0.000	1.000	0.000	1.000
OTU_133	0.000	1.000	0.000	1.000	0.000	1.000	0.000	1.000
OTU_134	0.000	1.000	0.000	1.000	-0.061	0.798	0.000	1.000
OTU_135	0.000	1.000	0.000	1.000	0.000	1.000	0.000	1.000
OTU_136	0.000	1.000	0.000	1.000	0.000	1.000	0.000	1.000
OTU_137	0.289	0.216	0.000	1.000	-0.106	0.657	-0.216	0.361
OTU_138	0.000	1.000	0.000	1.000	0.000	1.000	0.000	1.000
OTU_139	0.000	1.000	0.000	1.000	0.000	1.000	0.000	1.000
OTU_140	0.000	1.000	0.000	1.000	0.000	1.000	0.230	0.329
OTU_141	0.000	1.000	0.000	1.000	0.000	1.000	0.000	1.000
OTU_142	0.158	0.505	0.146	0.539	0.407	0.075	**0.642**	**0.002**
OTU_143	-0.119	0.619	0.000	1.000	-0.285	0.223	0.000	1.000
OTU_144	0.140	0.556	-0.172	0.469	0.047	0.843	0.102	0.670
OTU_145	0.000	1.000	0.000	1.000	0.007	0.977	0.400	0.080
OTU_146	0.000	1.000	0.000	1.000	-0.312	0.181	-0.004	0.987
OTU_147	0.000	1.000	0.000	1.000	0.000	1.000	0.000	1.000
OTU_148	0.000	1.000	0.000	1.000	0.000	1.000	0.000	1.000
OTU_149	0.000	1.000	0.000	1.000	0.000	1.000	0.000	1.000
OTU_150	0.000	1.000	0.000	1.000	0.000	1.000	0.000	1.000
OTU_151	0.000	1.000	0.000	1.000	0.000	1.000	0.000	1.000
OTU_152	0.000	1.000	0.000	1.000	0.000	1.000	0.000	1.000
OTU_153	0.000	1.000	0.000	1.000	0.000	1.000	0.000	1.000
OTU_154	0.000	1.000	0.000	1.000	0.000	1.000	0.000	1.000
OTU_155	0.000	1.000	0.000	1.000	0.000	1.000	0.000	1.000
OTU_156	0.000	1.000	0.000	1.000	0.000	1.000	0.000	1.000
OTU_157	0.229	0.332	0.000	1.000	0.000	1.000	0.000	1.000
OTU_158	0.000	1.000	0.000	1.000	0.000	1.000	0.000	1.000
OTU_159	0.000	1.000	0.000	1.000	0.000	1.000	0.000	1.000
OTU_160	0.000	1.000	0.000	1.000	0.000	1.000	0.000	1.000
OTU_161	0.000	1.000	0.000	1.000	0.000	1.000	0.000	1.000
OTU_162	0.000	1.000	0.000	1.000	0.000	1.000	0.000	1.000
OTU_163	0.000	1.000	0.000	1.000	0.000	1.000	0.000	1.000
OTU_164	0.000	1.000	0.000	1.000	0.000	1.000	**0.667**	**0.001**
OTU_165	0.000	1.000	0.000	1.000	0.000	1.000	0.000	1.000
OTU_166	0.000	1.000	0.000	1.000	0.000	1.000	0.330	0.155
OTU_167	0.000	1.000	0.000	1.000	0.000	1.000	0.000	1.000
OTU_168	0.000	1.000	0.000	1.000	0.000	1.000	0.000	1.000
OTU_169	0.000	1.000	0.000	1.000	0.000	1.000	0.000	1.000

OTU_170	0.000	1.000	0.000	1.000	0.000	1.000	0.000	1.000
OTU_171	0.000	1.000	0.000	1.000	0.000	1.000	0.424	0.063
OTU_172	-0.272	0.246	-0.289	0.216	-0.368	0.111	0.000	1.000
OTU_173	0.000	1.000	0.000	1.000	0.000	1.000	0.000	1.000
OTU_174	0.000	1.000	0.000	1.000	0.000	1.000	0.000	1.000
OTU_175	0.000	1.000	0.000	1.000	0.000	1.000	0.000	1.000
OTU_176	0.000	1.000	0.000	1.000	0.000	1.000	0.000	1.000
OTU_177	0.000	1.000	-0.161	0.498	0.372	0.106	0.000	1.000
OTU_178	0.000	1.000	0.000	1.000	0.000	1.000	0.000	1.000
OTU_179	0.000	1.000	0.000	1.000	0.000	1.000	0.000	1.000
OTU_180	0.000	1.000	0.000	1.000	0.000	1.000	0.106	0.657
OTU_181	0.000	1.000	0.000	1.000	0.000	1.000	0.000	1.000
OTU_182	0.000	1.000	0.106	0.657	-0.273	0.244	0.000	1.000
OTU_183	0.118	0.620	0.346	0.135	0.137	0.565	-0.003	0.990
OTU_184	0.000	1.000	0.000	1.000	-0.312	0.181	0.000	1.000
OTU_185	0.000	1.000	0.000	1.000	0.000	1.000	0.000	1.000
OTU_186	0.000	1.000	0.000	1.000	0.000	1.000	0.000	1.000
OTU_187	0.000	1.000	0.000	1.000	0.000	1.000	0.000	1.000
OTU_188	0.000	1.000	0.000	1.000	0.000	1.000	0.000	1.000
OTU_189	0.000	1.000	0.000	1.000	0.000	1.000	0.000	1.000
OTU_190	0.000	1.000	0.000	1.000	0.000	1.000	0.000	1.000
OTU_191	0.000	1.000	0.000	1.000	0.000	1.000	0.000	1.000
OTU_192	0.000	1.000	0.000	1.000	0.000	1.000	0.000	1.000
OTU_193	0.000	1.000	0.000	1.000	0.000	1.000	0.000	1.000
OTU_194	0.000	1.000	0.000	1.000	0.000	1.000	0.000	1.000
OTU_195	0.000	1.000	0.000	1.000	0.000	1.000	0.000	1.000
OTU_196	0.000	1.000	0.000	1.000	0.000	1.000	0.000	1.000
OTU_197	0.000	1.000	0.000	1.000	0.000	1.000	0.000	1.000
OTU_198	0.000	1.000	0.000	1.000	0.084	0.725	0.000	1.000
OTU_199	0.000	1.000	0.000	1.000	0.000	1.000	0.000	1.000
OTU_200	0.000	1.000	0.000	1.000	0.000	1.000	0.000	1.000
OTU_201	0.000	1.000	0.000	1.000	0.000	1.000	0.000	1.000
OTU_202	0.241	0.307	0.000	1.000	0.417	0.067	0.000	1.000
OTU_203	0.000	1.000	0.000	1.000	0.000	1.000	0.000	1.000
OTU_204	0.000	1.000	0.000	1.000	0.000	1.000	0.000	1.000
OTU_205	0.000	1.000	0.000	1.000	0.000	1.000	0.000	1.000
OTU_206	0.000	1.000	0.000	1.000	0.000	1.000	0.000	1.000
OTU_207	0.000	1.000	0.000	1.000	0.000	1.000	0.000	1.000
OTU_208	-0.067	0.780	-0.262	0.264	-0.010	0.966	-0.103	0.667
OTU_209	0.000	1.000	0.000	1.000	0.000	1.000	0.000	1.000
OTU_210	0.000	1.000	0.000	1.000	0.000	1.000	0.000	1.000
OTU_211	0.000	1.000	0.000	1.000	0.000	1.000	0.000	1.000
OTU_212	0.000	1.000	0.000	1.000	0.000	1.000	0.000	1.000

OTU_213	0.000	1.000	0.000	1.000	0.000	1.000	0.000	1.000
OTU_214	0.000	1.000	0.000	1.000	0.000	1.000	0.417	0.067
OTU_215	0.000	1.000	0.000	1.000	0.000	1.000	0.000	1.000
OTU_216	0.000	1.000	0.000	1.000	0.000	1.000	0.000	1.000
OTU_217	0.000	1.000	0.000	1.000	0.000	1.000	0.000	1.000
OTU_218	0.000	1.000	0.000	1.000	0.000	1.000	0.000	1.000
OTU_219	0.000	1.000	0.000	1.000	0.046	0.848	0.000	1.000
OTU_220	0.000	1.000	0.000	1.000	-0.059	0.804	0.394	0.085
OTU_221	0.000	1.000	0.000	1.000	0.000	1.000	0.000	1.000
OTU_222	0.000	1.000	0.000	1.000	0.000	1.000	0.000	1.000
OTU_223	0.000	1.000	0.000	1.000	0.000	1.000	0.000	1.000
OTU_224	0.000	1.000	0.000	1.000	0.000	1.000	0.000	1.000
OTU_225	0.000	1.000	0.000	1.000	0.000	1.000	0.180	0.447
OTU_226	0.000	1.000	0.000	1.000	0.000	1.000	0.000	1.000
OTU_227	0.000	0.999	0.000	1.000	0.109	0.649	0.000	1.000
OTU_228	0.000	1.000	0.000	1.000	0.000	1.000	0.000	1.000
OTU_229	0.000	1.000	0.000	1.000	0.000	1.000	0.000	1.000
OTU_230	-0.279	0.234	0.000	1.000	0.000	1.000	0.000	1.000
OTU_231	0.000	1.000	0.000	1.000	0.000	1.000	0.000	1.000
OTU_232	0.000	1.000	0.000	1.000	0.000	1.000	0.000	1.000
OTU_233	0.000	1.000	0.000	1.000	0.000	1.000	0.000	1.000
OTU_234	0.000	1.000	0.000	1.000	0.000	1.000	0.000	1.000
OTU_235	0.000	1.000	0.000	1.000	0.000	1.000	0.000	1.000
OTU_236	0.000	1.000	0.000	1.000	0.000	1.000	0.000	1.000
OTU_237	0.031	0.898	0.000	1.000	-0.312	0.181	0.000	1.000
OTU_238	0.000	1.000	0.000	1.000	0.000	1.000	0.000	1.000
OTU_239	0.000	1.000	0.000	1.000	0.000	1.000	0.000	1.000
OTU_240	0.000	1.000	0.000	1.000	0.000	1.000	0.000	1.000
OTU_241	0.000	1.000	0.000	1.000	0.391	0.088	0.000	1.000
OTU_242	0.000	1.000	0.000	1.000	0.000	1.000	-0.175	0.462
OTU_243	0.000	1.000	0.000	1.000	0.000	1.000	0.000	1.000
OTU_244	0.000	1.000	0.000	1.000	0.000	1.000	0.000	1.000
OTU_245	0.000	1.000	0.000	1.000	0.000	1.000	0.000	1.000
OTU_246	0.000	1.000	0.000	1.000	0.000	1.000	0.000	1.000
OTU_247	0.000	1.000	0.000	1.000	0.000	1.000	0.000	1.000
OTU_248	0.256	0.276	0.000	1.000	0.000	1.000	0.000	1.000
OTU_249	0.000	1.000	0.000	1.000	0.000	1.000	0.000	1.000
OTU_250	0.000	1.000	0.000	1.000	0.000	1.000	0.000	1.000
OTU_251	0.000	1.000	0.000	1.000	-0.222	0.347	0.000	1.000
OTU_252	0.000	1.000	0.000	1.000	0.000	1.000	0.000	1.000
OTU_253	0.000	1.000	0.000	1.000	0.375	0.103	0.169	0.478
OTU_254	0.000	1.000	0.000	1.000	0.034	0.886	-0.002	0.992
OTU_255	0.000	1.000	0.000	1.000	0.000	1.000	0.000	1.000

OTU_256	0.000	1.000	0.000	1.000	0.000	1.000	0.176	0.459
OTU_257	0.000	1.000	0.000	1.000	0.000	1.000	0.000	1.000
OTU_258	0.000	1.000	0.000	1.000	0.000	1.000	0.000	1.000
OTU_259	0.001	0.997	0.314	0.177	0.000	1.000	-0.076	0.749
OTU_260	0.000	1.000	0.000	1.000	0.000	1.000	0.000	1.000
OTU_261	0.000	1.000	0.000	1.000	0.000	1.000	0.000	1.000
OTU_262	0.000	1.000	0.000	1.000	0.000	1.000	0.000	1.000
OTU_263	0.000	1.000	0.000	1.000	0.391	0.088	0.000	1.000
OTU_264	0.000	1.000	0.000	1.000	0.000	1.000	0.000	1.000
OTU_265	0.000	1.000	-0.289	0.216	0.080	0.738	0.000	1.000
OTU_266	0.000	1.000	-0.063	0.791	0.000	1.000	0.000	1.000
OTU_267	0.000	1.000	0.000	1.000	0.000	1.000	0.000	1.000
OTU_268	0.000	1.000	0.000	1.000	0.000	1.000	0.000	1.000
OTU_269	0.000	1.000	0.000	1.000	0.000	1.000	0.000	1.000
OTU_270	-0.176	0.459	0.000	1.000	0.000	1.000	0.176	0.459
OTU_271	-0.063	0.791	0.000	1.000	0.000	1.000	0.000	1.000
OTU_272	0.000	1.000	0.000	1.000	0.000	1.000	0.000	1.000
OTU_273	0.000	1.000	0.000	1.000	0.000	1.000	0.000	1.000
OTU_274	0.000	1.000	0.000	1.000	0.000	1.000	0.000	1.000
OTU_275	0.000	1.000	-0.063	0.791	0.000	1.000	0.000	1.000
OTU_276	0.000	1.000	0.000	1.000	0.000	1.000	0.000	1.000
OTU_277	0.000	1.000	0.000	1.000	0.000	1.000	0.000	1.000
OTU_278	0.000	1.000	0.000	1.000	0.000	1.000	0.000	1.000
OTU_279	0.000	1.000	0.000	1.000	0.000	1.000	0.000	1.000
OTU_280	0.000	1.000	0.000	1.000	0.000	1.000	0.000	1.000
OTU_281	0.016	0.948	0.000	1.000	0.000	1.000	**0.458**	**0.042**
OTU_282	0.000	1.000	0.000	1.000	0.000	1.000	0.000	1.000
OTU_283	0.000	1.000	0.000	1.000	0.000	1.000	0.000	1.000
OTU_284	0.000	1.000	0.000	1.000	0.000	1.000	0.000	1.000
OTU_285	0.000	1.000	0.000	1.000	0.000	1.000	0.000	1.000
OTU_286	0.025	0.917	0.000	1.000	0.000	1.000	0.000	1.000
OTU_287	0.024	0.920	-0.298	0.201	0.181	0.446	0.043	0.856
OTU_288	0.000	1.000	0.000	1.000	0.000	1.000	0.000	1.000
OTU_289	0.000	1.000	0.000	1.000	0.000	1.000	0.000	1.000
OTU_290	0.000	1.000	0.000	1.000	0.000	1.000	0.000	1.000
OTU_291	0.000	1.000	0.000	1.000	0.000	1.000	0.000	1.000
OTU_292	0.000	1.000	0.000	1.000	0.000	1.000	0.000	1.000
OTU_293	0.000	1.000	0.000	1.000	0.086	0.717	0.000	1.000
OTU_294	0.000	1.000	0.000	1.000	0.000	1.000	0.000	1.000
OTU_295	0.000	1.000	0.000	1.000	0.000	1.000	0.077	0.747
OTU_296	0.000	1.000	0.000	1.000	0.000	1.000	0.000	1.000
OTU_297	0.000	1.000	-0.044	0.853	0.000	1.000	0.000	1.000
OTU_298	0.000	1.000	0.000	1.000	0.000	1.000	0.000	1.000

OTU_299	0.000	1.000	0.000	1.000	0.000	1.000	0.000	1.000
OTU_300	0.000	1.000	-0.268	0.253	**0.669**	**0.001**	0.000	1.000
OTU_301	0.167	0.482	0.000	1.000	0.000	1.000	0.000	1.000
OTU_302	0.000	1.000	0.000	1.000	**0.559**	**0.010**	0.394	0.085
OTU_303	0.000	1.000	0.000	1.000	0.000	1.000	0.000	1.000
OTU_304	0.000	1.000	0.000	1.000	0.000	1.000	0.356	0.124
OTU_305	-0.050	0.835	-0.211	0.372	0.330	0.155	0.000	1.000
OTU_306	0.000	1.000	0.000	1.000	0.000	1.000	0.000	1.000
OTU_307	0.000	1.000	0.000	1.000	-0.289	0.216	0.000	1.000
OTU_308	0.000	1.000	0.000	1.000	0.000	1.000	0.000	1.000
OTU_309	0.000	1.000	0.000	1.000	0.232	0.324	0.000	1.000
OTU_310	0.000	1.000	0.000	1.000	0.000	1.000	0.000	1.000
OTU_311	0.000	1.000	0.000	1.000	0.000	1.000	0.000	1.000
OTU_312	0.000	1.000	0.000	1.000	0.000	1.000	0.000	1.000
OTU_313	0.000	1.000	0.000	1.000	0.000	1.000	0.400	0.081
OTU_314	0.000	1.000	0.000	1.000	0.000	1.000	0.000	1.000
OTU_315	0.000	1.000	0.000	1.000	0.000	1.000	0.000	1.000
OTU_316	0.000	1.000	0.000	1.000	0.000	1.000	0.000	1.000
OTU_317	0.000	1.000	0.000	1.000	0.000	1.000	0.000	1.000
OTU_318	0.000	1.000	0.000	1.000	0.000	1.000	0.000	1.000
OTU_319	0.000	1.000	0.000	1.000	0.000	1.000	0.000	1.000
OTU_320	0.000	1.000	0.000	1.000	0.000	1.000	0.000	1.000
OTU_321	0.000	1.000	0.000	1.000	0.000	1.000	0.000	1.000
OTU_322	0.000	1.000	0.000	1.000	0.000	1.000	-0.064	0.789
OTU_323	0.000	1.000	0.000	1.000	0.000	1.000	0.000	1.000
OTU_324	0.000	1.000	0.000	1.000	0.000	1.000	0.000	1.000
OTU_325	0.000	1.000	0.000	1.000	0.000	1.000	0.000	1.000
OTU_326	0.000	1.000	0.000	1.000	0.000	1.000	0.000	1.000
OTU_327	0.000	1.000	0.000	1.000	0.000	1.000	0.000	1.000
OTU_328	0.000	1.000	0.000	1.000	0.000	1.000	0.391	0.088
OTU_329	0.000	1.000	0.000	1.000	0.000	1.000	0.000	1.000
OTU_330	0.000	1.000	0.000	1.000	0.000	1.000	0.000	1.000
OTU_331	0.000	1.000	0.000	1.000	0.000	1.000	0.000	1.000
OTU_332	0.000	1.000	0.000	1.000	0.000	1.000	0.000	1.000
OTU_333	0.000	1.000	0.000	1.000	0.391	0.088	0.000	1.000
OTU_334	0.000	1.000	0.000	1.000	0.000	1.000	0.000	1.000
OTU_335	0.000	1.000	0.000	1.000	0.417	0.067	-0.400	0.081
OTU_336	0.000	1.000	0.000	1.000	0.000	1.000	0.000	1.000
OTU_337	0.000	1.000	0.000	1.000	0.000	1.000	0.000	1.000
OTU_338	0.000	1.000	0.000	1.000	0.000	1.000	0.000	1.000
OTU_339	0.000	1.000	0.000	1.000	0.000	1.000	0.000	1.000
OTU_340	0.000	1.000	0.000	1.000	0.000	1.000	0.000	1.000
OTU_341	0.000	1.000	0.000	1.000	-0.004	0.987	0.000	1.000

OTU_342	0.000	1.000	0.000	1.000	0.000	1.000	0.000	1.000
OTU_343	0.000	1.000	0.000	1.000	0.000	1.000	0.000	1.000
OTU_344	0.000	1.000	0.000	1.000	-0.141	0.553	0.000	1.000
OTU_345	0.000	1.000	0.000	1.000	0.000	1.000	0.000	1.000
OTU_346	0.000	1.000	0.000	1.000	0.000	1.000	0.000	1.000
OTU_347	0.000	1.000	0.000	1.000	0.000	1.000	0.000	1.000
OTU_348	0.000	1.000	0.000	1.000	0.000	1.000	0.000	1.000
OTU_349	0.222	0.347	0.000	1.000	0.000	1.000	0.000	1.000
OTU_350	0.000	1.000	0.000	1.000	0.000	1.000	0.000	1.000
OTU_351	0.000	1.000	0.000	1.000	0.000	1.000	0.000	1.000
OTU_352	0.000	1.000	0.000	1.000	-0.004	0.987	0.000	1.000
OTU_353	0.000	1.000	0.000	1.000	0.000	1.000	0.000	1.000
OTU_354	0.000	1.000	0.000	1.000	0.000	1.000	0.000	1.000
OTU_355	0.000	1.000	0.142	0.550	0.000	1.000	0.000	1.000
OTU_356	0.000	1.000	0.000	1.000	0.000	1.000	0.420	0.065
OTU_357	0.000	1.000	0.000	1.000	0.000	1.000	0.000	1.000
OTU_358	0.000	1.000	0.000	1.000	0.000	1.000	0.000	1.000
OTU_359	0.000	1.000	0.000	1.000	0.000	1.000	0.000	1.000
OTU_360	0.000	1.000	0.000	1.000	0.000	1.000	0.000	1.000
OTU_361	0.000	1.000	0.000	1.000	0.000	1.000	0.000	1.000
OTU_362	0.000	1.000	0.000	1.000	0.000	1.000	0.000	1.000
OTU_363	0.000	1.000	0.000	1.000	0.000	1.000	0.000	1.000
OTU_364	0.000	1.000	0.000	1.000	0.000	1.000	0.000	1.000
OTU_365	0.000	1.000	0.000	1.000	0.312	0.181	0.000	1.000
OTU_366	0.000	1.000	0.000	1.000	0.000	1.000	0.000	1.000
OTU_367	0.000	1.000	0.000	1.000	0.000	1.000	0.000	1.000
OTU_368	0.000	1.000	0.000	1.000	0.000	1.000	0.000	1.000
OTU_369	0.000	1.000	0.000	1.000	0.000	1.000	-0.064	0.789
OTU_370	0.000	1.000	0.000	1.000	0.000	1.000	0.000	1.000
OTU_371	0.289	0.216	0.000	1.000	0.000	1.000	0.000	1.000
OTU_372	0.000	1.000	0.000	1.000	0.143	0.549	**0.487**	**0.030**
OTU_373	0.000	1.000	0.000	1.000	0.000	1.000	0.000	1.000
OTU_374	0.000	1.000	0.000	1.000	-0.391	0.088	0.000	1.000
OTU_375	0.000	1.000	0.000	1.000	0.000	1.000	0.000	1.000
OTU_376	0.000	1.000	0.000	1.000	0.000	1.000	0.000	1.000
OTU_377	0.000	1.000	0.000	1.000	0.000	1.000	0.000	1.000
OTU_378	0.000	1.000	0.000	1.000	0.000	1.000	0.000	1.000
OTU_379	-0.006	0.981	0.000	1.000	0.000	1.000	0.000	1.000
OTU_380	0.000	1.000	0.000	1.000	-0.391	0.088	0.000	1.000
OTU_381	0.000	1.000	0.000	1.000	0.000	1.000	0.000	1.000
OTU_382	0.000	1.000	0.000	1.000	0.000	1.000	0.000	1.000
OTU_383	0.000	1.000	0.000	1.000	0.276	0.239	0.000	1.000
OTU_384	0.307	0.188	0.013	0.957	0.046	0.849	-0.007	0.976

OTU_385	0.000	1.000	0.000	1.000	0.000	1.000	0.000	1.000
OTU_386	0.000	1.000	0.142	0.550	0.000	1.000	0.000	1.000
OTU_387	0.000	1.000	0.000	1.000	0.000	1.000	0.000	1.000
OTU_388	0.000	1.000	0.000	1.000	0.000	1.000	0.000	1.000
OTU_389	0.000	1.000	0.000	1.000	0.417	0.067	0.000	1.000
OTU_390	0.000	1.000	0.000	1.000	0.000	1.000	0.000	1.000
OTU_391	0.000	1.000	0.000	1.000	0.000	1.000	0.000	1.000
OTU_392	0.000	1.000	0.000	1.000	0.000	1.000	0.000	1.000
OTU_393	0.000	1.000	0.000	1.000	0.000	1.000	0.394	0.085
OTU_394	0.000	1.000	0.000	1.000	0.000	1.000	0.000	1.000
OTU_395	0.000	1.000	0.000	1.000	0.000	1.000	0.000	1.000
OTU_396	**-0.476**	**0.034**	-0.327	0.159	-0.280	0.231	-0.170	0.475
OTU_397	0.000	1.000	0.000	1.000	0.000	1.000	0.000	1.000
OTU_398	0.000	1.000	0.000	1.000	0.000	1.000	0.000	1.000
OTU_399	0.000	1.000	0.000	1.000	0.000	1.000	0.000	1.000
OTU_400	0.000	1.000	0.000	1.000	0.000	1.000	0.000	1.000
OTU_401	0.000	1.000	0.000	1.000	-0.117	0.624	0.000	1.000
OTU_402	0.000	1.000	0.000	1.000	0.000	1.000	0.000	1.000
OTU_403	0.000	1.000	0.000	1.000	0.391	0.088	0.000	1.000
OTU_404	0.000	1.000	0.000	1.000	0.000	1.000	0.000	1.000
OTU_405	0.000	1.000	0.000	1.000	0.000	1.000	0.000	1.000
